# Plant-derived exosome-like nanovesicles: mechanisms and molecular understanding in neurological disorders with potential therapeutic applications

**DOI:** 10.1007/s13346-025-01955-0

**Published:** 2025-08-20

**Authors:** Sevim Isik, Sedra Alhelwani, Aya Sahsahi, Hilal Balcilar, Bercem Yeman-Kiyak

**Affiliations:** 1 Department of Molecular Biology and Genetics, Faculty of Engineering and Natural Sciences, Uskudar University, Uskudar, Istanbul 34662 Türkiye; 2 Stem Cell Research and Application Center (USKOKMER), Uskudar University, Uskudar, Istanbul 34662 Türkiye; 3 NPCELLAB Cellular Therapy, Stem Cell and Advanced Therapy Medicinal Products GMP Laboratory, Üsküdar University, Istanbul, Türkiye; 4 Department of Molecular Biology, Institute of Science, Uskudar University, Uskudar, Istanbul 34662 Türkiye

**Keywords:** Alzheimer's disease, Engineered exosomes, Neurological disorders, Parkinson's disease, Personalized medicine, Plant-derived exosome-like nanovesicles

## Abstract

Exosomes are nano vesicles secreted by the cells that play an essential role in intercellular communication, enabling the transport of bioactive molecules, including proteins, lipids, and nucleic acids. Among them, plant-derived exosome-like nanovesicles have attracted considerable interest due to their prospective therapeutic implications, especially for neurological disorders. This article provides an overview of the biogenesis of plant-derived exosome-like nanovesicles, compares their characteristics with mammalian-derived exosomes, and investigates their bioavailability and chemical composition. The article also discusses the mechanisms through which they are uptaken by cells, highlighting several cellular uptake pathways and their significance for targeted drug delivery. Moreover, it explains the molecular basis of neurological disorders and investigates how plant-derived exosome-like nanovesicles regulate intracellular signaling pathways, providing potential therapeutic benefits. Finally, it provides the latest advancements in engineering research, emphasizing biochemical modifications on the exosomal surface, loading therapeutic molecules into exosomes, and exosomes derived from genetically engineered plants, for more effective therapies in neurological disorders.

**Graphical abstract:**

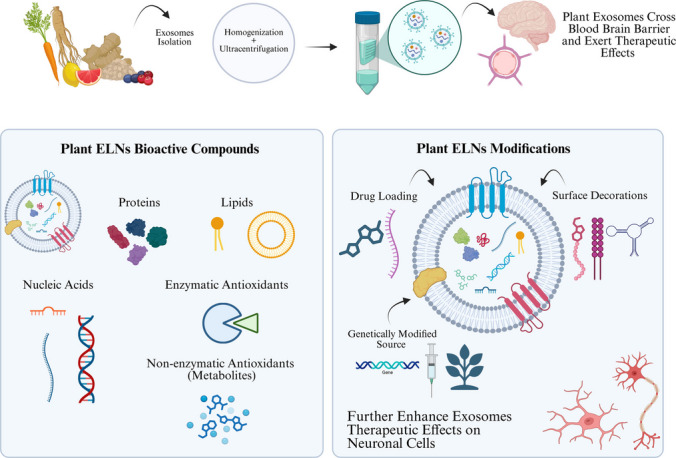

## Introduction

Exosomes are small nano-scaled membrane-bound vesicles secreted by eukaryotic cells and have gained significant attention for their potential as therapeutic vehicles, particularly in neurological disorders [1]. Plant-derived exosome-like nanovesicles (ELNs), which are naturally occurring nanoparticles secreted from plant cells, have emerged as a promising alternative to mammalian-derived exosomes and conventional nanocarriers. They have several benefits rendering them appeal for neurological therapy. Plant ELNs can cross the blood–brain barrier (BBB), have minimal immunogenicity, and are inherently rich in bioactive substances, including antioxidants and small RNAs. They exhibit biocompatibility, cost-effectiveness, and suitability for oral or nasal administration, which is atypical for traditional delivery methods. They are scalable and morally advantageous, overcoming manufacturing and safety limits associated with mammalian-derived vesicles. These characteristics establish plant ELNs as versatile tools for therapeutic administration and modulation of neuroinflammatory and neurodegenerative pathways [2]. The biogenesis of these ELNs involves the formation of intraluminal vesicles within multivesicular bodies (MVBs), which are released into the extracellular environment upon fusion with the plasma membrane, encapsulating proteins, lipids, DNAs, RNAs, and metabolites [3]. This makes them ideal for targeted drug delivery and intercellular communication. One of the key advantages of plant-derived ELNs is their superior biostability compared to mammalian exosomes, largely due to their robust lipid membranes, enabling them to withstand environmental stresses and maintain their integrity in gastric and intestinal fluids [4]. This stability is crucial for overcoming challenges such as the ability to cross the BBB, which often limits the delivery of therapeutic agents to the central nervous system (CNS), and to withstand gastric fluid [5–7].

Neurological disorders are a highly diversified category of conditions including Alzheimer's Disease (AD), Parkinson's Disease (PD), spinal cord injury (SCI), ischemic brain injuries, and brain tumors, which are all characterized by complex pathologies involving neuronal degeneration, neuroinflammation, oxidative stress, and impaired cellular communication in CNS. These disorders may result from genetic, environmental, and immunological factors, resulting in complex pathological processes. Many neurological disorders, despite their diversity, exhibit common molecular processes such as neuroinflammation, oxidative stress, mitochondrial dysfunction, and synaptic abnormalities. Furthermore, neurodegeneration, glial cell activation, and compromised neural transmission are fundamental to the pathophysiology of these disorders [8, 9].

Various current strategies are being explored to treat neurological disorders, including the use of nanogels and liposomes. Although these methods can deliver drugs and support neuronal regeneration, several significant challenges persist. For instance, nanogels may struggle to penetrate deeply into the brain tissues and might cause issues such as burst drug release, biocompatibility concerns, and neurotoxicity [10]. Liposomes may accumulate in non-target tissues because of their limited specificity for brain regions, potentially leading to long-term toxicity. Additionally, large liposome particles often have difficulty in crossing the BBB. They also exhibit low bioavailability, poor water solubility and scalability issues, all of which can diminish their effectiveness in treating certain brain areas [11]. Moreover, Long Non-Coding RNAs (lncRNAs) have been explored for their therapeutic potential in neurological disorders, owing to their role as gene regulators. However, their application remains constrained by challenges such as off-target delivery risks and immunogenic responses induced by synthetic RNA molecules [12, 13]. Therefore, there is an urgent need to develop highly specific delivery systems capable of preventing unintended interactions and reaching deep brain structures for long-term treatment—potential solutions include natural delivery vehicles or plant-derived ELNs.

Plant-derived ELNs have emerged as alternative promising therapeutic agents against many disorders, demonstrating their ability to modulate inflammatory pathways, reduce oxidative stress, regulate autophagy, and promote neuroprotection. Notably, plant-derived ELNs exhibit antioxidant, anti-inflammatory, antimicrobial and anti-cancer properties [14, 15]. Also, these ELNs play a critical role in targeting pro-inflammatory proteins which are linked with neuroinflammation, for instance, ginger rhizomes-derived ELNs, have shown anti-inflammatory properties by inhibiting NLRP3 inflammasome activation in macrophages, suggesting plant-derived ELNs could target proinflammatory proteins involved within relative signaling pathways in other neurological diseases [16]. These findings highlight the considerable potential of plant ELNs in therapeutic applications, from cancer and aging to gut health and inflammation. Alongside this, surface modifications of plant-derived ELNs enhance their targeting specificity, stability, and ability to cross the BBB. The functionalization of plant-derived ELN surfaces involves PEGylation [17], which is the insertion of maleimide lipid group, to act as bridges and connect with other targeting ligands e.g., peptides (RVG, RGD and aptamers), and coating of small molecules like folic acid, all of which improve ELNs targeting and interaction with various specific receptors of targeted cells, ensuring the therapeutic cargo reaches the desired site of action [18]. These modifications also reduce exosome immunogenicity, making them suitable for long-term therapies without eliciting unwanted immune responses [19].

This review seeks to provide an in-depth exploration of plant-derived ELNs, focusing on their biogenesis, stability, cellular pathways associated with neurological disorders, the signaling pathways which exosomes have the potential to regulate in neurological disorders, and recent advancements in engineered exosomes such as surface-decorated, cargo-loaded and source-based modified (transgenic) exosomes and their interactions with neuronal and glial cells in the brain, to enhance their therapeutic potential in treating complex neurological diseases.

## Biogenesis and comparative aspects of plant-derived ELNs

### Biogenesis

**Fig. 1:**
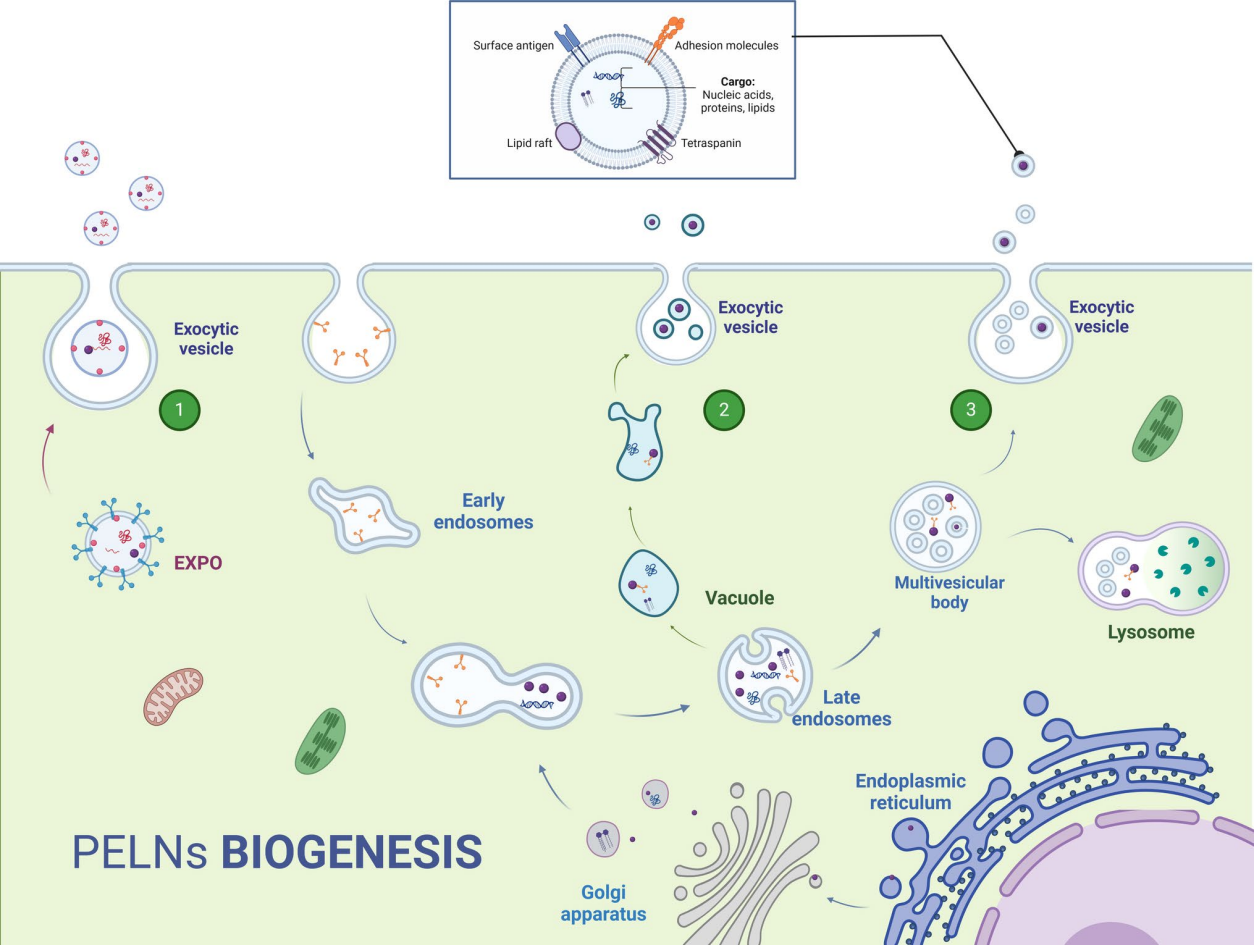
Biogenesis of plant-derived ELNs

Exosomes are small membrane-bound vesicles with sizes ranging from 30–200 nm. They are composed of a unique content of bioactive compounds enclosed in a lipid bilayer. Exosomes biogenesis starts with the plasma membrane invagination to form early endosomes which then mature into late endosomes [20]. The late endosomes undergo a transformation where their membrane folds inward to form smaller vesicles known as intraluminal vesicles (ILVs) within the endosome. As a whole, the endosome is now called a multivesicular body (MVB). Selectively, on the other hand, a unique combination of DNA, RNA, proteins, and lipids is gathered in the ILVs within MVBs. After formation, the MVBs face two fates, either fusing with a lysosome and getting degraded, or fusing with the cell's plasma membrane and releasing the ILVs extracellularly as free exosomes [3, 21]. The biogenesis of exosomes in plants can also take an alternative pathway. Double membrane organelles known as EXocyst Positive Organelles (EXPOs) have been identified in some plants like tobacco. They are similar to autophagosomes except that they are not associated with any degradation pathway, instead, they are involved in a direct secretion pathway [22] (Fig. 1).

### Cross-species comparison of exosomes features

While mammalian exosomes have been extensively studied and despite their biocompatibility, biodistribution, and low immune response, they have faced many challenges such as low stability, homogeneity issues, high cost and low mass production. On the other hand, plant-derived ELNs exhibit intriguing characteristics of higher stability, non-toxicity, uniformity, lower cost and higher mass production, all of which have shed light on their usage in nanomedicine research [2, 20]. These plant ELNs contain phospholipids, glycolipids, sterols, and polysaccharides with high phospholipids levels due to the lack of cholesterol, unlike mammalian exosomes. The presence of cellulose, hemicellulose, and pectin in plant cell walls provides additional protection, enhancing the stability and resilience of plant ELNs against harsh conditions. Their unique phytochemical composition includes polyphenols, phenolic acids, flavonoids, carotenoids, terpenoids, and alkaloids, pool in fostering dietary antioxidants intake, increasing cellular health and metabolic balance [23].

### Bioavailability

Studies reveal that certain plant extracts, such as onion powder, can inhibit inflammatory pathways, though solubility and bioavailability issues persist [24]. Recently, researchers have noted that even processed plant materials retain extracellular vesicles, which may offer nutritional and therapeutic benefits beyond fresh intake [25].

Free antioxidants, while crucial for alleviating oxidative stress and providing therapeutic and preventive effects against aging, cancer, neurodegenerative disorders, and cardiovascular disease, still confront several difficulties that restrict their stabilities, ranging from inadequate bioavailability, poor stability, and ineffective targeting. Vitamin C, for example, declines during juice processing due to its susceptibility to oxygen and temperature, and because of the intestinal wall's low permeability, necessitating its conversion to dehydroascorbic acid [26]. Similarly, crocins as in extracts face challenges in crossing the in vitro blood–brain barrier (BBB) (PAMPA assay) in their free form due to their relatively large size and susceptibility to degradation. However, crocins may be able to penetrate the BBB if they are metabolized into a simpler form, specifically deglycosylated trans-crocetin, although this process occurs at a slow rate [27]. Therefore, encapsulation into exosomes provides novel delivery strategies to these limitations. Exosomes not only protect their cargo from destruction, but they also allow for effective delivery to specific organs, including the brain, via routes such as the gut-brain axis. Citrus lemon exosomes, for example, increase vitamin C stability and provide safe delivery while avoiding interference with blood coagulation pathways [28]. Similarly, tomafran-derived exosomes naturally enriched with crocins and picrocrocin pass the BBB and reduce oxidative stress and inflammation more effectively than free antioxidants [27]. This encapsulating approach thus opens a promising path for dietary supplements and therapeutic treatments, particularly in neurodegenerative illnesses such as AD and PD.

### Chemical components of plant-derived ELNs

Besides the unique composition, plant-derived ELNs have rich proteomic and lipidomic profiles, making them significant for intercellular communication and potential therapeutic applications.

**Fig. 2:**
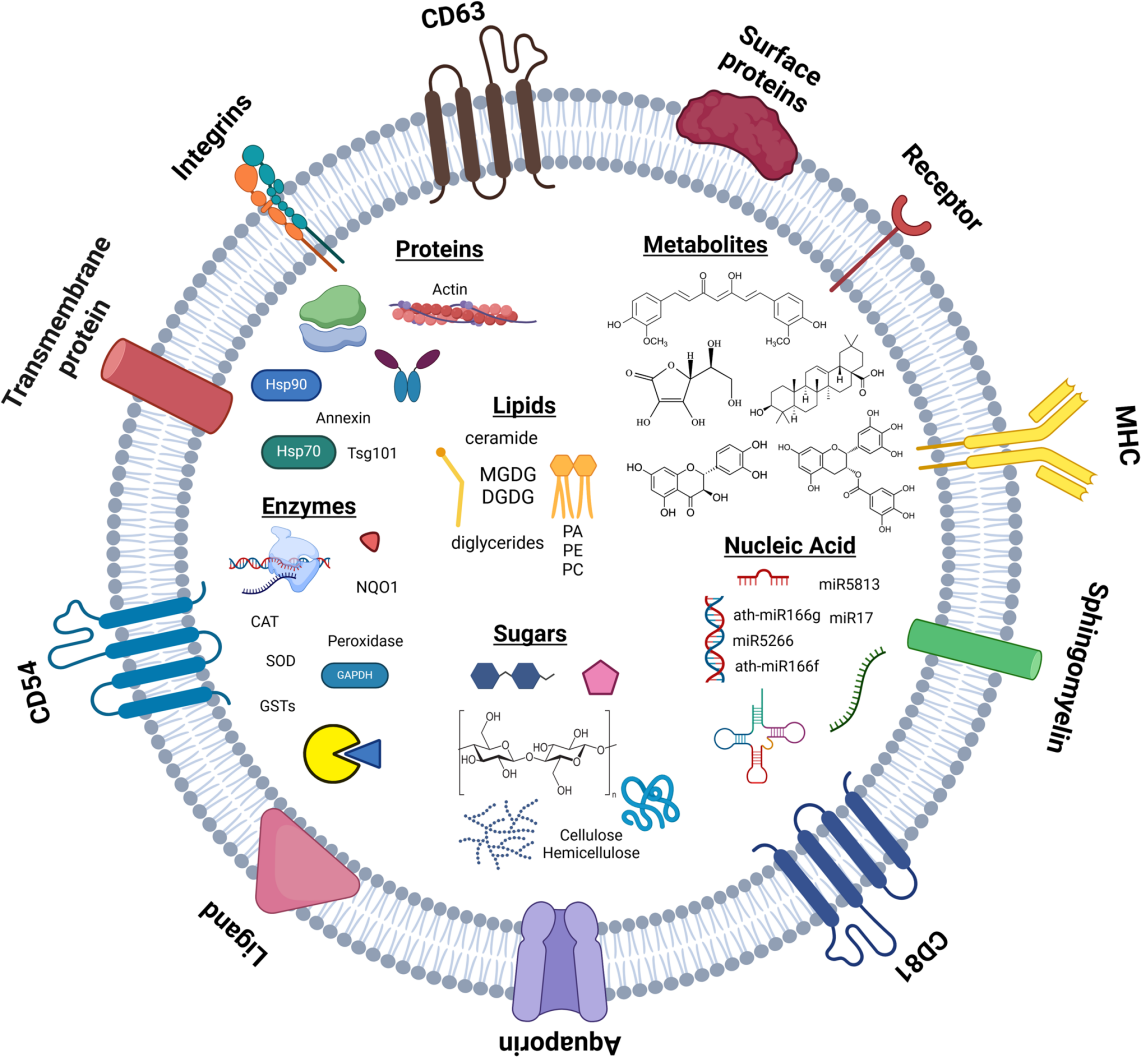
Structural characteristic of plant-derived ELNs

General structural characteristics of Plant-derived ELNs are demonstrated in Fig. 2. Most of the protein content in plant ELNs transmembrane and cytosolic proteins among which are proteolysis, aquaporin, and proteases in addition to channels and transporters [29, 30]. They also contain proteins such as glutathione S-transferase, S-adenosyl-homocysteinase, glyceraldehyde 3 phosphate dehydrogenase, annexins, and heat shock proteins or tetraspanins, which are strong candidates to be accepted as plant ELNs markers (e.g., CD9, CD63, CD81, and CD54) [31].

Not only their protein content but also the lipid content plays crucial roles in cellular responses and intercellular communication. They maintain exosome stability and structure, facilitate cargo uptake and retention. Studies have shown that plant ELNs acquire higher concentrations of essential components like phospholipids (such as phosphatidic acids (PA), phosphatidylethanolamines (PE), mono- and di-galactosyldiacylglycerol (e.g., MGDG, DGDG)).

miRNAs, non-coding, single-stranded molecules with length around 21 to 23 nucleotides found in eukaryotic cells [32]. Plant miRNAs are specific to plants and plant activities, but lately, it has been found that when ingested and absorbed by animal cells, it may alter the expression of some of the host organism’s genes [33]. Another group of researchers found evidence of plant miRNA in circulating blood of human subject [34].

## Cellular uptake mechanisms of plant-derived ELNs

Plant-derived ELNs are internalized by cells via a variety of cellular uptake mechanisms. Understanding these mechanisms is crucial, as they elucidate how these vesicles deliver their diverse cargo, including RNA, proteins, and lipids to target cells. Cellular uptake pathways and surface interactions are critical determinants of their therapeutic potential, particularly in neurological disorders. Cellular uptake mechanisms can be broadly divided into endocytic pathways, surface interactions, and nanovesicle composition [20, 35, 36].

### Endocytic pathways

Endocytosis refers to the process by which cells internalize extracellular materials through the invagination of the plasma membrane. Various pathways, including clathrin-mediated endocytosis, caveolin-mediated endocytosis, macropinocytosis, and phagocytosis, are involved. These pathways effectively deliver plant-derived ELN cargo to target cells [36, 37]. Clathrin-mediated endocytosis involves the recruitment of clathrin proteins to form coated vesicles, internalizing specific cargoes. ELNs may be recognized and internalized by membrane-bound receptors [38, 39]. Receptor-mediated endocytosis ensures selective uptake, triggering vesicle formation and intracellular transport [40]. Caveolin-mediated endocytosis involves lipid-rich invaginations and interactions with membrane components. This pathway avoids lysosomal degradation, preserving structural integrity for cytosolic delivery [41]. Macropinocytosis engulfs extracellular fluid and its contents nonspecifically. Phagocytosis internalizes larger particles, often by immune cells [41]. The endocytic pathway utilized depends on ELN size, composition, and surface properties [38]. Pathway selection influences ELN-based therapeutic effectiveness [23].

### Interactions of surface proteins between cells and exosomes

Surface proteins on plant-derived ELNs, such as tetraspanins, integrins, and glycoproteins, selectively bind receptors on target cells, enabling receptor-mediated endocytosis. These interactions ensure efficient uptake and intracellular trafficking [42, 43]. Specific proteins may prevent lysosomal degradation, facilitating cargo delivery to therapeutic targets [41]. Selective interactions between ELN surface proteins and receptors, like hippocalcin, enable precise targeting for neurological therapies [44–46]. Lipids and glycoproteins further enhance selective binding and uptake [23]. Modifications to ELN surfaces improve targeting capabilities and therapeutic efficacy [47]. Lipids, such as sphingolipids, influence membrane fusion and transport. These modifications impact uptake mechanisms and distribution, facilitating targeted delivery [6, 7, 36]. Researchers are actively exploring surface engineering to improve biodistribution and therapeutic potential.

### Impact of ELN composition on uptake mechanism

Size, lipid profile, and protein composition impact internalization pathways and therapeutic applications of plant-derived ELNs [48]. Those that are larger in size, favor macropinocytosis or phagocytosis, while smaller ones utilize clathrin- or caveolin-mediated pathways [49]. Lipids, like phosphatidylserine, enhance recognition and internalization by phagocytic cells [50]. Surface proteins and glycoproteins trigger receptor-mediated endocytosis, facilitating efficient cargo delivery [35]. Researchers are investigating these features to develop targeted therapies [51]. Plant-derived ELNs contain bioactive compounds, like gingerol or proanthocyanidins, that influence cellular uptake and therapeutic potential [44–46]. Understanding these factors is essential for optimizing these ELNs as drug-delivery systems and therapeutic agents.

## Cellular mechanisms in neurological disorders

### Alzheimer’s disease

AD is a progressive neurodegenerative disorder characterized by key pathological hallmarks, including synaptic dysfunction due to tau protein hyperphosphorylation, reduced neuronal metabolism, and the loss of multiple neurotransmitters such as acetylcholine, noradrenaline, serotonin, and dopamine. A major structural issue is the disruption of microtubules, caused by hyperphosphorylated tau proteins that destabilize them, leading to neuronal breakdown [52]. AD is marked by extracellular amyloid beta (Aβ) accumulation, a byproduct of abnormal amyloid precursor protein (APP) processing via β-secretase and γ-secretase, and intracellular neurofibrillary tangles formed from tau protein. Aβ binds to degenerated axons, apolipoprotein E, microglia, and astrocytes, impairing synaptic signaling and inducing inflammation [53, 54]. Monoamine oxidase (MAO) plays a role by breaking down neurotransmitters like dopamine, serotonin, and noradrenaline, producing hydrogen peroxide which diffuses through the cell membrane, elevating oxidative stress and promoting lipid peroxidation, which damages neuron structures, triggering inflammatory cytokine release [55]. Oxidative stress also exacerbates Aβ and tau tangle aggregation, creating a feedback loop of increasing free radicals. High reactive oxygen species (ROS) levels further damage cellular components, like mitochondria, leading to disrupted Ca^2+^ homeostasis through N-methyl-D-aspartate (NMDA) receptor overactivation and endoplasmic reticulum (ER) Ca^2+^ overload [56]. Elevated intracellular Ca^2+^ influx triggers pathways such as calcineurin and Bcl-2-associated death promoter activation, releasing cytochrome c, which initiates apoptosis. Aβ also interacts with caspases, breaking down essential cell components and leading to neuronal death [57]. Additionally, ROS activates pathways like stress-activated protein kinase (JNK) and p38 mitogen-activated protein kinase (MAPK), both of which hyperphosphorylate tau, contributing to neurofibrillary tangle formation which in turn leads to alterations in neuronal signaling and neuronal loss especially in the hippocampus [58], resulting in significant acetylcholine depletion due to reduced choline acetyltransferase activity [59]. Overall, the accumulation of Aβ plaques and neurofibrillary tangles, along with oxidative stress and neuroinflammation, forms a vicious cycle, disrupting neuronal function and communication, eventually resulting in memory and cognitive decline.

Current treatments in AD are commonly targeted towards easing the symptoms and retarding disease advancement rather than curing it, as there is none. Cholinesterase inhibitors and glutamate regulators are mostly used as the pharmacological approach [60]. However, although debated, monoclonal antibodies targeting the amyloid plaques have also been a recent option of treatment with ongoing clinical trials [61, 62]. On the other hand, non-pharmacological approaches such as cognitive stimulation, exercise, and diet are also included in the treatment protocol in order to improve patients’ quality of life [63–65]. Even though these therapies have improved, challenges still remain. These include side effects, high expenses, accessibility difficulties, limited slowing of disease progression due to late diagnosis, and individual variability. Therapies with personalized methods are needed in order to improve patient outcomes. In this sense, stem cell and exosomal therapies have become prominent to promote brain regeneration and repair.

### Parkinson’s disease

PD is a neurodegenerative disorder marked by the loss of dopaminergic neurons in the substantia nigra, a brain region responsible for producing dopamine. The disease manifests through motor symptoms affecting movement and balance and non-motor symptoms including pain, sleep problems and mental health issues [66, 67]. The death of dopaminergic neurons reduces dopamine levels in the synapses and leads to the formation of Lewy bodies (LB), primarily composed of aggregated alpha-synuclein (α-Syn) [68]. These abnormal protein deposits also contain neurofilament proteins and proteins involved in cellular breakdown, like ubiquitin. The main cause of cell death in PD is thought to be the breakdown of the nuclear membrane, releasing aggregated α-Syn, which interacts with nuclear elements such as histones [69]. α-Syn is primarily found in presynaptic terminals in the CNS, where it binds to synaptic vesicles [70]. α-Syn contains an amphipathic N-terminus, which helps it bind lipids and potentially aids in aggregation. Its non-amyloid component at the C-terminus binds calcium and inhibits aggregation [71]. The protein aggregates, known as amyloids, consist of misfolded proteins rich in β-sheets, which are associated with cellular damage and neuronal dysfunction. Amyloid deposition is linked to issues such as mitochondrial dysfunction, oxidative stress, and disruptions in the ER and proteasomes, contributing to neuronal degeneration [69]. Currently, there is no cure for PD. The standard treatment involves dopaminergic medications like levodopa, COMT inhibitors, anticholinergics, dopamine agonists, and MAO-B inhibitors, which aim to manage symptoms and improve patients'mobility and life expectancy [68]. However, these treatments provide only symptomatic relief without addressing the disease's root causes, highlighting the need for a more comprehensive approach to PD treatment.

### Brain tumors (cancer mechanisms)

Brain cancer, also known as a CNS tumor, is one of the most aggressive and challenging forms of cancer to treat. It is basically a group of abnormal cell growth, can be benign or malignant, that originates within the brain or the spinal cord. The main types of brain cancer include gliomas, meningiomas, and pituitary tumors, each with distinct characteristics and prognoses. Gliomas, which originate from glial cells, are the most common type of brain cancer, accounting for over 80% of all malignant brain tumors [72]. The biological and molecular basis of brain cancer is complex, involving genetic mutations and alterations in signaling pathways that drive uncontrolled cell growth and proliferation [73]. Furthermore, recent research has highlighted the significance of the HGF/MET (Hepatocyte growth factor/Mesenchymal-epithelial transition factor) signaling pathway in the pathogenesis and progression of malignant brain tumors [74]. Treatments for brain cancer have historically been limited, with surgical resection, radiation therapy, and chemotherapy being the primary options. However, there are limitations in the effectiveness of these standard treatments, as brain tumors can often be resistant to therapy and can recur even after aggressive treatment [75]. Newly emerging treatment options, such as targeted molecular therapies, immunotherapy, and personalized medicine approaches, hold promise in improving outcomes for patients with brain cancer [76, 77]. Targeted therapies aim to disrupt specific molecular pathways involved in cancer progression, while immunotherapy seeks to harness the body's own immune system to recognize and attack tumor cells [78]. Additionally, personalized medicine approaches, which involve genetic profiling of individual tumors, can guide the selection of the most effective treatment regimen for each patient. Despite these advances, the challenge of treating brain cancer remains significant, as the BBB can limit the delivery and efficacy of these novel therapies [79, 80]. However, plant exosome-based delivery methods are innovative approaches to overcoming the BBB that are currently being explored to improve the delivery of therapeutic agents to brain tumors. These exosome-based delivery systems have shown promising results in preclinical studies, demonstrating enhanced permeability across the BBB and targeted delivery of therapeutic payloads to brain tumor cells [81, 82].

### Ischemic brain injury

Ischemic stroke, a progressive and life-threatening condition, accounts for 85% of all stroke cases. It occurs when an artery in the brain becomes blocked, cutting off the oxygen-rich blood supply to the brain, known as cerebral arterial occlusion. This blockage is typically caused by either a clot forming directly in the brain (thrombosis) or one that forms elsewhere and travels to the brain (embolism) [83, 84]. The resulting damage unfolds through a cascade of events that progressively deteriorate brain tissue. The initial trigger is the loss of oxygen and glucose due to blood flow obstruction, which leads to hypoxia in neurons. This deprives them of ATP, as oxidative phosphorylation in the mitochondria halts, resulting in an energy failure. Consequently, harmful substances such as glutamate, ROS, and reactive nitrogen species (RNS) are released, exacerbating oxidative stress. As Na⁺ levels rise, causing an influx of calcium ions (Ca^2^⁺), triggering excitotoxicity, which further damages cells. The excessive oxidative stress also stimulates the release of pro-inflammatory cytokines, such as TNF-α, IL-6, and IL-1β, which trigger an inflammatory response and activate microglia and macrophages. As ischemia continues, the tissue becomes more acidic causing nearby neurons to depolarize, extending the injury into the peri-infarct region. This cascade inhibits neuronal repair and leads to apoptosis. In areas where ATP depletion is severe, necrosis occurs, while autophagy may either protect or contribute to further cell death depending on the severity of the ischemia. Prolonged ischemia also damages the BBB, allowing immune cells, proteins, and other substances to leak into the brain, which accelerates inflammation and causes brain swelling (edema) [83]. The current gold standard treatment for ischemic stroke, recombinant tissue plasminogen activator (rt-PA), was approved by the FDA in 1996. Administered intravenously, rt-PA is effective by dissolving the clot obstructing blood flow. However, its limitations were witnessed when it was not administered within the time window, delays in administration can lead to complications such as hemorrhagic transformation, neurotoxicity, and increased mortality [85, 86].

### Spinal cord injury

Spinal cord injury (SCI) is a severe neurological condition arising from primary injury of both traumatic causes—such as falls and road traffic accidents—and nontraumatic factors, including infections and tumors, which could all lead to glial membrane and axonal network damage as well as immediate damage to neuronal tissues. As the population ages, the incidence of SCIs, particularly from falls, is expected to rise [87]. In the secondary injury phase, SCI triggers a cascade of complex, interconnected pathological changes at the injury site due to excessive glutamate, which causes neuronal excitotoxicity with calcium (Ca^2^⁺) and sodium ions (Na⁺) influx, ionic imbalances, inflammation and necrosis. First, ischemia and hypoxia initiate edema and mitochondrial dysfunction, reducing cellular energy and ATP and inducing alterations in the morphology of neuronal cells [88]. This leads to an overproduction of ROS and RNS, causing widespread damage to cell membranes by mediating lipid peroxidation, proteins, and DNA. As oxidative stress intensifies, pro-inflammatory cytokines are released, creating a vicious cycle of inflammation that stimulates microglia activation and macrophages infiltration. These processes contribute to glial scar formation, further hindering recovery, increasing tissue necrosis, and decreasing neuronal regeneration, ultimately leading to extensive neuronal apoptosis. The result is paralysis, disruptions in sensory function, and even potential organ failure due to the extent of damage to the nervous system [87, 89]. After SCI, several factors limit tissue regeneration at the lesion core. Non-neuronal cells (fibroblasts, microglia, endothelial cells) contribute to the formation of a fibrotic scar. Similarly, astrocytes form an astroglial scar, which acts as a barrier to protect the surrounding tissues from further damage, although it also hinders regeneration [87]. Recent research indicates that commonly used anti-inflammatory such as corticosteroids and neurotrophic medications in SCI treatment do not adequately target early oxidative stress. Therefore, they are ineffective in significantly improving SCI symptoms [90, 91]. While surgery can help improve motor function in traumatic SCIs, it often results in complications like respiratory and cardiovascular problems [92]. Despite medical advancements, there is still no effective drug to reduce the intense oxidative stress following SCI, highlighting a significant gap in current treatment options [93].

### Gut-brain axis-related disorders

The gut-brain axis refers to the bidirectional communication between the gastrointestinal tract and the CNS, which plays a crucial role in regulating various physiological and neurological functions. This intricate network involves neural, endocrine, and immune signaling pathways that allow the gut microbiota to influence brain function and vice versa. The four major pathways of the gut-brain axis include the vagus nerve, the neuroendocrine system, the immune system, and the gut microbiota-derived metabolites and signaling molecules [94, 95]. Dysregulation of this axis has been linked to a range of neurological and psychiatric disorders, including AD, PD, multiple sclerosis (MS), autism spectrum disorder, depression, and anxiety [96]. PD, for instance, can be triggered by gut microbiota dysbiosis and inflammation [97]. Conversely, AD has been associated with gut microbiome alterations that may contribute to neuroinflammation and the development of pathological hallmarks in the brain [98]. In MS, gut dysbiosis and intestinal barrier dysfunction can lead to increased permeability and the entry of inflammatory molecules into CNS, exacerbating autoimmune responses [99]. Moreover, the gut-brain axis has been implicated in the pathogenesis of autism spectrum disorder, with disruptions in the microbiome-gut-brain signaling potentially contributing to the core symptoms of the condition [100]. Similarly, depression and anxiety have been linked to imbalances in the gut microbiome, which can influence neurotransmitter production, neural plasticity, and the stress response [101]. The key symptoms and signs of gut-brain axis-related disorders often include gastrointestinal issues, cognitive impairments, mood disturbances, and neurological deficits. These diseases typically progress through stages of gut dysbiosis, increased intestinal permeability, neuroinflammation, and the accumulation of pathogenic proteins, leading to the manifestation of clinical symptoms [102, 103]. The biological and molecular basis of these disorders involves complex interactions between the gut microbiome, the immune system, and the CNS, with ongoing research aimed at elucidating the precise mechanisms underlying these connections [104]. Key mechanisms include the production of neuroactive metabolites, the modulation of the immune system, and the regulation of the hypothalamic–pituitary–adrenal axis, which governs the body's response to stress. These pathways can influence neurotransmitter synthesis, neuronal excitability, synaptic plasticity, and brain-derived neurotrophic factor expression, all of which play critical roles in neurological and psychiatric conditions [105, 106]. Current treatment options for gut-brain axis-related disorders include dietary interventions, probiotics, antibiotics, anti-inflammatory medications, and neurotransmitter-targeting therapies, but their effectiveness is often limited by the complex and multifactorial nature of these disorders [96]. Challenges in effectively treating these diseases include the need for personalized approaches, the difficulty in modulating the gut microbiome, and the lack of a comprehensive understanding of the precise mechanisms linking the gut and the brain [107]. Nonetheless, promising discoveries about plant-derived ELNs offer hope for the development of more effective treatments in the future.

## Intracellular signaling pathways modulated by plant-derived ELNs in neurological disorders and their therapeutic potential

Neurological disorders such as neurodegenerative diseases including AD and PD, as well as acute injuries, such as strokes, have complex, interrelated molecular mechanisms specific to them. The most crucial pathways involved in the progression of these diseases include neuroinflammation, neuroprotection and synaptic plasticity, autophagy and protein homeostasis, antioxidant and calcium signaling pathways [9]. The operational flow of these pathways is necessary to maintain healthy, functional and responsive neurons against oxidative stress, damage or inflammation. In recent studies, it has been shown that plant-derived ELNs have a therapeutic potential in neurological disorders through modulating the above-mentioned pathways. Accumulating more knowledge about the regulation and overlapping of these pathways can shed light on possible therapeutic approaches for various neurological disorders.

### Neuroinflammation pathways

Neuroinflammation is a series of interwind and complicated cascade of events that happens in the central nervous system and it can be triggered by injuries, exposure to toxins or infection. The detection of the previously mentioned risks leads to the upregulation of inflammation signaling pathways including NF-κB, MAPK, and JAK/STAT pathways. Microglial cells are then activated leading to elevated secretion of pro-inflammatory cytokines, chemokines, ROS and nitric oxide [9]. This reaction is initially protective but in a chronic state it contributes to the progression of neurological diseases. Studies revealed that *Allium Tubersum* and *Atractylodes lancea* ELNs (A-ELNs and ALR-ELNs respectively) both exhibit anti-inflammatory effects by inhibiting inducible nitric oxide synthase (iNOS) expression, and reducing pro-inflammatory cytokines [108, 109]. Another notable study reported that exosomes isolated from *Lycium Barbarum L.* when loaded with isoliquiritigenin (ISL), significantly impact the neuroinflammatory pathway by reducing pro-inflammatory cytokines like IL-1β, IL-6, TNF-α, while increasing the expression of anti-inflammatory cytokine IL-10. Additionally, it inhibits the expression of iNOS, and activates the pAKT/AKT pathway which is critical for survival. Lastly, it promotes neuroprotection and synaptic plasticity by upregulating Tuj1, MAP2, GAP43, NF200, and MBP markers [110–112].

### Antioxidant signaling pathways

The Antioxidant signaling pathway is a cellular defense mechanism against elevated ROS levels. The detection and response to oxidative stress involve a wide network of genes, enzymes, and many regulatory molecules [113]. The core regulator of this pathway in the CNS is the nuclear factor erythroid 2–related factor 2 (Nrf2), which controls the production of key enzymes including heme oxygenase-1 (HO-1) and superoxide dismutase (SOD) [114]. It is responsible for maintaining redox homeostasis and neutralizing ROS, which eventually protects the cells from oxidative damage [115]. In this context, the previously mentioned ALR-ELNs also promote neuroprotection through pathways involving immunoresponsive gene 1 (IRG1) and Nrf2 [109]. *Drynariae rhizoma* ELNs target oxidative phosphorylation and mitochondrial dysfunction in Huntington's disease, while modulating Nrf2/ARE signaling to provide antioxidant and neuroprotective benefits [116]. *Lycium Reuthenicum* ELNs (LRM-ELNs) reduce oxidative stress by enhancing nuclear Nrf2 translocation and boosting HO-1 and NQO1 expression in an in vitro AD model [117, 118]. Furthermore, a study showed that nanoparticles derived from carrot reduce 6-OHDA-induced oxidative stress and apoptosis in SH-SY5Y cells by suppressing caspase-3 cleavage and modulating Nrf-2, Heme oxygenase (HO-1) as well as NAD(P)H Quinone Dehydrogenase 1 (NQO-1) [119]. Onion-derived exosomes also provided protection against oxidative stress by activating KEAP1/NRF2/ARE signaling pathway, reducing ROS levels by enhancing HO-1 expression and translocation of Nrf-2 [110–112].

### Neuroprotection and apoptosis

The brain's apoptosis and neuroprotection pathways are two contrasting but related biological mechanisms that regulate neuronal survival and death in response to a range of pathological and physiological events [120]. Key neuroprotective pathways include the MAPK/ERK, PI3K/Akt, and Nrf2 signaling cascades, while apoptosis is regulated through intrinsic (mitochondrial) and extrinsic (death receptor) pathways. Both pathways eventually converge on caspase-3 activation [121]. A recent study shows that LRM-ELNs reduce Aβ-induced apoptosis in PC12 cells by downregulating MAPK and enhancing the PI3K/Akt pathway [117, 118]. In another study, researchers showed that *Pueraria Lobata* Roots derived exosomes enhance PINK1-Parkin-mediated mitophagy and promote SQSTM1/p62-mediated autophagy [122]. Additional research reported that LRM-ELNs maintain mitochondrial membrane potential (MMP) and lower the Bax/Bcl-2 ratio in the setting of Aβ-induced apoptosis in HT22 cells. This lowers Caspase-3 cleavage and, ultimately, inhibits the intrinsic apoptotic pathway [117, 118].

### Calcium signaling

Calcium (Ca2 +) is a very important molecule in the CNS. It works as a secondary molecule that regulates many neuronal processes such as synaptic transmission, gene expression, plasticity, and cell survival through various calcium signaling pathways [123]. Glutamate excitotoxicity on the other hand is a hallmark of various neurological diseases and is mediated by dysregulation of calcium signaling pathway [124]. In addition to iron dysregulation and lipid peroxidation, high glutamate causes an increase in intracellular Ca2 +, which in turn leads to ferroptosis. To mitigate this, a group of researchers proved that green onion-derived ELNs can reduce lipid peroxidation by enhancing the expression of glutathione peroxidase 4 (GPX4) and inhibit glutamate-induced Ca2 + influx in HT22 mouse hippocampal cells [125].

Mechanisms and molecular insights of plant-derived exosomes in neurological disorders is detailed in Table 1.

**Table 1:** Mechanisms and molecular insights of plant-derived exosomes in neurological disorders

Phyto-exosomes source	Isolation Method	Size (nm)	Zeta Potential (mV)	Identified Bioactive Components	Therapuetic Potential	Study Type	Disease Targets	Mechanisms of Action/Effects	Ref
Citrus Lemon	UC	93.77 ± 21.31 nm (DLS)	−3.46 ± 1.45 mV	Vitamin C	Antioxidant Neuroproctective	In vitro (Neuroblastoma: SH-SY5Y)	AD	↑ Antioxidant activity ↓ Aβ-induced neurotoxicity Ability to cross BBB (PAMPA assay) Maintain neuronal health	[28]
Tomafran (Bioengineered Tomato)	UC	169 nm (NTA)	-	Corcins	Antioxidant Anti-inflammatory Neuroproctective	In vitro (Neuroblastoma: SH-SY5Y)	AD	↑ Neuroprotection ↓ Oxidative stress and inflammation ↓ Okadaic acid-induced tau hyperphosphorylation	[27]
Lycium Ruthenicum Murray	PEG6000-based precipitation	114.1 nm (NTA)	−6.36 mV	Polysaccharides Polyphenols Anthocyanins	Antioxidant Antiapoptotic Neuroproctective	In vitro (Pheochromocytoma cells: PC12)	AD	↓ Aβ-induced apoptosis ↓ MAPK ↑ PI3K/AKT pathway ↓ Bax ↑ Bcl-2	[117, 118]
Lycium Ruthenicum Murray	PEG6000-based precipitation	114.1 nm (NTA)	−6.36 mV	-	Antioxidant Antiapoptotic Neuroproctective	In vitro (Mouse hippocampal neuronal cells: HT22)	AD	↓ Aβ-induced apoptosis ↑ MMP ↓ Bax/↑ Bcl-2 ↓ Caspase-3 cleavage ↓ Oxidative stress ↑ NRF-2 ↑ HO-1 ↑ NQO1	[117, 118]
Salvia Sclarea Hairy Roots	UC + SEC	116 ± 15 nm (NTA)	-	Triterpenoids: asiatic acid, ursolic acid, oleanolic acid, and peroxidases	Neuroprotective Antioxidant Anti-apoptotic ​	In vitro (Neuroblastoma: SH-SY5Y)	PD	↓ 6-OHDA autoxidation and ROS ↓ Apoptosis ↑ Mitochondrial function Maintain metabolic homeostasis	[126]
Pueraria Lobata Roots	UC + MF	125.0 ± 9.7 nm (DLS)	−5.0 ± 0.7 mV	Puerarin, daidzein, genistein, and miRNAs: hsa-miR-16-5p, hsa-miR-30a-5p, hsa-let-7d-5p, hsa-miR-15b-5p	Antioxidant Anti-inflammatory Neuroprotective	In vitro (Neuroblastoma: SH-SY5Y) and In Vivo (C57BL/6 mice)	PD	↑ PINK1-Parkin- + SQSTM1/p62-mediated mitophagy ↑ MMP ↓ Oxidative stress ↓ LRRK2 expression Maintains MRC complexes I and V Restore ATP synthase	[122]
Carrots	SEC + UF	143.9 nm (NTA)	−10.2 mV	-	Antioxidative Anti-apoptotic	In vitro (Neuroblastoma: SH-SY5Y)	PD	↓ 6-OHDA-induced oxidative stress ↓ Apoptosis ↓ Caspase-3 cleavage ↑ NRF-2 ↑ HO-1 ↑ NQO1	[119]
Drynariae Rhizoma Root	dUC + 0.22 μm MF	71.67 nm (NanoFCM)	-	NAD(P)H-quinone oxidoreductase, DNA-directed RNA polymerases, ribosomal proteins, ATP synthase, maturase K, phytochrome	Antioxidant Neuroprotective	Proteomics + Bioinformatics	AD, PD, HD	Activate Nrf2/ARE axis crucial for cellular defense Involvement of oxidative phosphorylation could help in: ↓ Oxidative stress ↓ Mitochondrial dysfunction Maintain neuronal health	[116]
Green Onion	PEG8000-based precipitation	167.4 nm (DLS)	− 16.06 mV	-	Antioxidant Antiferroptic Neuroproctective	In vitro (Mouse hippocampal neuronal cells: HT22)	Ferroptosis	↓ Glutamate-induced ferroptosis ↓ Oxidative stress ↓ Lipid peroxidation ↓ DMT1 and TfR1 ↑ FPN1 ↑ GPX4 Restore iron homeostasis and neuronal health	[125]
Onions	UC	156 nm (NTA)	−24.8 mV	-	Antioxidant Anti-inflammatory Antiapoptotic Neuroproctective	In vitro (Pheochromocytoma cells: PC12, Mouse microglial cells: BV2) In vivo (C57BL/6 mice)	SCI	Activate KEAP1/NRF2/ARE signaling pathway crucial for cellular defense ↓ Oxidative stress ↓ Apoptosis ↑ NRF-2 ↑ HO-1 ↑ SOD ↓ Caspase-3 cleavage ↓ Bax/↑ Bcl-2	[110–112]
Lycium Barbarum L	UC	151.45 ± 3.86 nm (DLS)	− 5.34 ± 0.472 mV	Isoliquiritigenin	Antioxidant Anti-inflammatory Antiapoptotic Neuroproctective Neuroregeneration	In vitro (Neural stem cells: NSCs and microglial cells: N9) In vivo (Rat SCI model, 3D-printed scaffold)	SCI	↓ iNOS ↓ TNF-α, ↓ IL-6, ↓ IL-1β ↑ IL-10 ↓ Oxidative stress Activate pAKT/AKT signaling pathway to promote axonal repair Promote neurogenesis by: ↑ Tuj1, ↑ MAP2, ↑ GAP43, ↑ NF200, ↑ MBP	[110–112]
Momordica Charantia	UC	131.6 nm (NTA)	-	miR5266	Anti-inflammatory Antiapoptotic Neuroproctective	In vitro (Mouse hippocampal neuronal cells: HT22) In vivo (Male Sprague–Dawley rats)	Ischemic Brain Injury	↓ MMP-9 Maintain the integrity of BBB Upregulate tight junction proteins: ↑ claudin-5, ↑ ZO-1 Activate PI3K/AKT/GSK3β signaling pathway crucial for cell survival ↓ Apoptosis ↓ Caspase-3 cleavage ↓ Bax/↑ Bcl-2	[127]
Panax Notoginseng	UC	151.3 nm (DLS)	−8 mV	Lipids ceramide, phosphatidic acid, diglycerides, polyunsaturated fatty acids, Proteins (206 cytosolic and plasma membrane proteins), miRNAs	Antioxidant Anti-inflammatory Neuroproctective	In vitro (Primary mouse microglia: BV2) In vivo (Male Sprague–Dawley rats)	Ischemic Reperfusion Injury	Ability to alter the microglia polarization: ↓ M1 phenotype (CD86 + CD206 −) ↑ M2 phenotype (CD86 − CD206 +) ↓ TNF-α, ↓ IL-6, ↑ IL-10 ↓ Oxidative stress ↓ Apoptosis Maintain BBB integrity Activate PI3K/Akt signaling pathway crucial for cell survival	[44–46]
Momordica charantia	UC	40–150 nm (NTA)	-	miRNAs e.g., miR-5266	Antioxidant Anti-inflammatory Neuroproctective	In vitro (Mouse brain microvescular endothelial cells: bEnd.3) In vivo (Male Sprague–Dawley rats)	Ischemic Stroke	Prevent ONOO⁻/HMGB1/MMP-9 Signaling Pathway ↓ oxidative stress ↓ Hemorrhagic Transformation ↑ Microvascular Integrity Upregulate tight junction proteins: ↑ claudin-5, ↑ ZO-1	[110–112]
Atractylodes lancea	UC + exoEasy Maxi Kit	50–365 nm (TEM)	-	Methoxycoumarin miRNAs: ath-miR166f, ath-miR162a-5p, ath-miR162b-5p	Antioxidant Anti-inflammatory Neuroproctective Neuroregeneration	In vitro (Primary mouse microglia: BV2)	Neuroinflamation	↓ iNOS ↓ TNF-α, ↓ IL-6, ↓ IL-1β, ↓ CCL2, ↓CXCL10 ↑ HO-1, ↑ IRF7 ↓ Oxidative stress Activate IRG1/NRF-2 essential for maintaining cellular homeostasis and brain's resilience	[109]
Allium Tubersum	UC + exoEasy Maxi Kit	1145 ± 2 nm (TEM)	-	Dexamethasone miRNAs: ath-miR166g, ath-miR168a-5p	Antioxidant Anti-inflammatory Neuroproctective	In vitro (Primary mouse microglia: BV2)	Neuroinflamation	↓ iNOS ↓ NO ↓ TNF-α, ↓ IL-6, ↑ HO-1, ↑ cGMP HO-1 produces CO and cGMP to mediate the anti-inflammatory and antioxidant effects	[108]
Oat	OptiPrep gradient purification	Exosomes were ranged 30–150 nm	-	β-glucan, digalactosyldiacylglycerol (DGDG), proteins, lipids, and polysaccharides	Antioxidant Anti-inflammatory Neuroproctective	In vitro (Primary mouse microglia: BV2) + In vivo (C57BL/6 mice)	Neuroinflamation	↓ Dectin-1-mediated inflammatory pathway ↓ TNF-α, ↓ IL-6, ↓ IL-1β ↓ Phospho-NF-κB ↓ Phospho-Syk Reduce neuronal apoptosis by: ↓ TNFR1, ↓ RIPK1 ↓ Caspases-3, −8 cleavage	[128]
Momordica Charantia	UC	120 nm (NTA)	-	miR5813	Anti-glioma Anti-metastatic Neuroprotective	In vitro (Human glioma cells: U251, T98G, U87; neuroblastoma: SH-SY5Y) + In vivo (BALB/c nude mice)	Gliomas	Inhibit the proliferation of U251 glioma cells Induce U251 glioma cells cycle arrest by: ↓ PCNA, ↓ Cyclin D1 ↑ P21 Suppress the migration and invasion of U251 glioma cells by: ↓ Vimentin, ↓ N-cadherin, ↓ MMP9 Prevent epithelial-mesenchymal transition (EMT) Inhibit pAKT/AKT signaling pathway Ability to cross BBB Accumulate in glioma tissues	[129]
Panax ginseng	UC	151.6 nm (NTA)	−17.9 mV	Ginsenosides (Rg1, Re, Rg3, and Rb1) miRNAs (98 types) and proteins (86 types)	Anti-glioma Anti-cancer Anti-inflammatory Neuroprotective	In vitro (Glioma: C6) + In vivo (male Wistar rat; Balb/C mice)	Gliomas	Ability to cross the BBB Ability to target glioma cells Exhibit non-cytotoxic effects Induce c-MYC gene silencing through ptc-miR396f Modulate Tumor Microenvironment by: ↑ M1 recruitment—↑ CD86, ↑ IL6 ↓ M2 polarization—↓ CD206, ↓ IL10 ↓ Tregs, ↓ CD8 + Regulate Cancer-Associated Fibroblasts by: ↓α-SMA, ↓ Integrins Reduce tumor progression mechanism by: ↓ TGF-β, ↓ IL-10 ↑ BAX, ↓ BCL-2, ↓ Survivin	[130]
Grapefruit	UC	135.3 ± 4.62 nm​ (DLS)	−37.7 ± 0.10 mV	Doxorubicin-loaded heparin-based nanoparticles (DNs)	Anti-glioma Antitumor Antiproliferation	In vitro (Glioma: LN229, U251, U87) + In vivo (BALB/c nude, C57BL/6 mice, Sprague–Dawley rats)	Gliomas	Ability to cross the BBB ↑ Invasion into tumor cells Mediate prolong the circulation time of drugs in the bloodstream ↑ IL-6, ↑ IFN-α, ↑ TNF-α, ↑ IP-10 Keep other levels of IL-1β, IL-2, or IFN-γ Reduce glioma cells proliferation by: ↓ Ki67, ↓ CD34	[131]
Citrus Lemon	UC	100.21 ± 3.35 nm (DLS)	-	DOX + Decorated with cRGD	Anti-inflammatory Neuroproctective Antitumor	In vitro (Mouse Glioblastoma: GL261; Mouse brain microvescular endothelial cells: bEnd.3) + In vivo (C57BL/6 J mice)	Gliomas	ELNs were engineered as structural droplets DOX-loaded “ESDDs” Ability to cross BBB and BBTB ↑ Invasion into tumor cells ↑ Intracellular Uptake ↑ Accumulation of DOX ↑ Glioblastoma chemotherapy ↓ Glioblastoma growth, ↓ Angiogenesis ↓ TNF-α, ↓ IL-6, ↓ IL-1β, ↓ IP-10 ↑ DOX bioavailability	[132]
Grapefruit	UC	87.2 ± 11.3 nm (DLS)	−13.9 mV	miR17	Anti-inflammatory Neuroproctective Antitumor	In vitro (Mouse glioblastoma: GL26-Luc) + In vivo (C57BL/6 J mice)	Brain Tumor	Deliver therapeutic miR17 to folate receptor-positive tumor cells ↓ MHCI on glioblastoma Activate NK cells Inhibit tumor growth Avoid immune activation by: ↓ F4/80 + macrophages ↓ Iba-1 + microglia	[133]
Garlic	UC	30–200 nm	-	Phosphatidic Acid (PA)	Antioxidant Anti-inflammatory Neuroproctective Anti-obesity	In vitro (Primary mouse microglia: BV2; Embryonic primary mouse neuronal cells: E17) + In vivo (C57BL/6 mice)	Obesity (gut/bain axis modulation)	↓ TNF-α, ↓ IL-6, ↓ IL-1β, ↓ IFN-γ Supress cGAS/STING inflammatory pathway ↓ MMP ↓ ROS ↓ Apoptosis ↓ IDO1 Enhance neurogenesis by: ↑ β-tubulin II, ↑ MAP2, ↑ Nestin, ↑ OCT4 Suppress the IDO1/AHR axis Reverse high-fat diet (HFD)-induced obesity and insulin resistance	[134]

UC: ultracentrifuge, dUC: differential ultracentrifuge, DSL: dynamic light scattering, NTA: nanoparticle tracking analysis, MF: membrane filtration, UF: ultrafiltration, TEM: transmission electron microscopy

## Engineered plant-derived ELNs

Engineered plant-derived ELNs represent a versatile and effective platform for delivering therapeutics to manage neurological disorders. Their inherent lipid composition confers biocompatibility, reducing toxicity and immunogenic concerns, thereby enabling repeated therapeutic applications. They can be engineered through two main approaches: post-isolation modifications, including surface functionalization and drug loading, as well as source-based modifications, such as genetically engineering transgenic plants to boost therapeutic potential [27]. Furthermore, these nanovesicles can be purposefully functionalized to enhance targeting specificity, improving precision. For instance, conjugation with DSPE-PEG-RVG augments selectivity towards dopaminergic neurons, facilitating receptor-mediated internalization and enabling treatments for conditions like PD [122]. Plant-derived ELNs can undergo advanced surface engineering through post-isolation modifications, including advanced surface functionalization, such as incorporating folic acid into grapefruit-derived ELNs to target folate receptor-positive tumor cells selectively. Hybridizing these ELNs with polyethylenimine (PEI) enhances RNA encapsulation efficiency while reducing the toxicity of free PEI [133]. Furthermore, employing hydrophobic insertion techniques and click chemistry allows for the addition of targeting ligands, like the R11-3 aptamer, to enhance cellular uptake by binding to specific receptors, such as those on BBB endothelial cells [19]. These surface modifications improve targeting specificity, cellular internalization, and therapeutic efficacy, solidifying plant-derived ELNs as a robust platform for precise drug delivery.

These nanovesicles are also engineered to cross the BBB, surmounting a key obstacle in neurological treatments. Their improved stability and extended circulation enhance bioavailability, while modifications bolster their cargo loading capacity [110–112]. The loading strategy for grapefruit-derived extracellular vesicles employs a biomimetic patching approach, integrating doxorubicin (DOX)-loaded heparin-based nanoparticles (DNs) onto the vesicle surface. This innovative method offers a fourfold increase in drug-loading capacity compared to traditional encapsulation techniques. Additionally, these engineered extracellular vesicle-drug nanoparticle hybrids exhibit pH-sensitive properties, enabling controlled release of DOX under acidic conditions, such as those found in tumor microenvironments. This ensures efficient delivery of the chemotherapeutic agent to glioma tissues, maximizing therapeutic efficacy while minimizing off-target effects [131]. Furthermore, these engineered vesicles enable the regulated release of their therapeutic payloads, facilitating sustained therapeutic impact. PDNVs exhibit immunocompatibility and facilitate the targeted delivery of biomolecules to diseased neurons, thereby modulating mitochondrial function and mitigating oxidative stress [108]. Furthermore, these versatile nanocarriers can encapsulate and transport therapeutic RNAs, proteins, and antioxidants, positioning them as a promising platform for revolutionizing the treatment of neurodegenerative disorders. Their safety, scalability, and non-invasive nature offer viable strategies for promoting brain health.

### Type of exosome modifications

#### Biochemical surface modifications

**Fig. 3:**
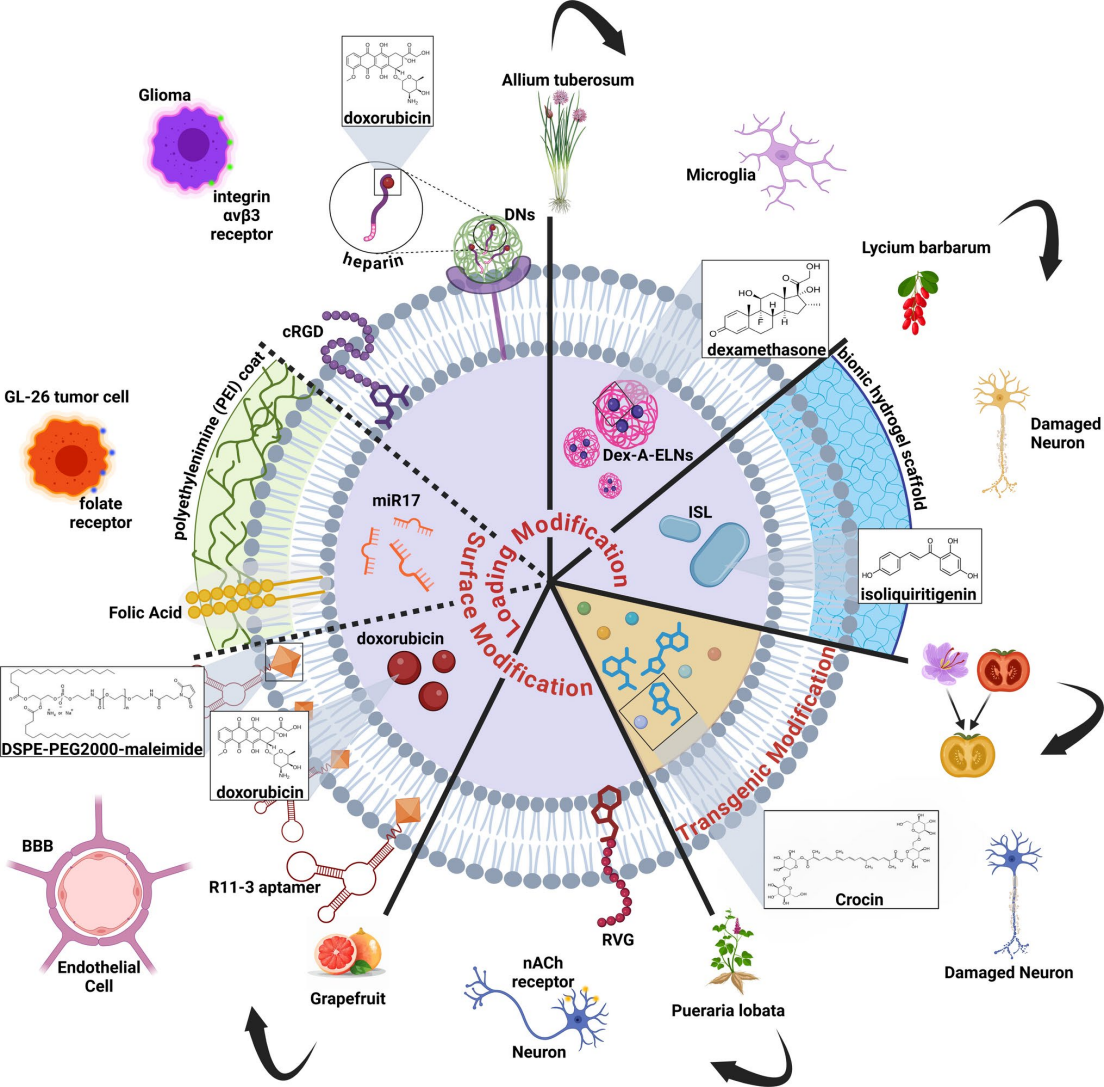
Post-isolation and source-based modifications of plant-derived ELNs

*Pueraria lobata*-derived ELNs, represent a novel and effective platform for delivering modified biomolecules for the treatment of neurodegenerative disorders [122]. The natural lipid bilayer structure and biocompatibility reduce toxicity and immunogenicity, allowing drugs to be used more efficiently and effectively. These molecules can cross BBB, allowing the delivery of biomacromolecules to diseased cells. Conjugation of Pu-ELNs membrane surface with ligands such as DSPE-PEG-RVG significantly increases their selectivity, especially for dopaminergic neurons, facilitating recognition and uptake by nicotinic acetylcholine receptors (nAChRs) (Fig. 3). Modification increases the transit time, reduces the immune response, and improves bioavailability, allowing for a long-lasting therapeutic effect. In a model of PD, Pu-ELNs have shown remarkable therapeutic potential by restoring mitochondrial function, reducing oxidative stress, and promoting PINK1-Parkin mitophagy, and reducing neurodegeneration [122].

Grapefruit-derived ELNs were surface-modified by incorporating a lipophilic folate ligand into their lipid bilayer during preparation [133]. This enabled the active targeting of folate receptor-positive tumor cells (Fig. 3). Additionally, the nano vectors were hybridized with PEI to enhance the efficiency of RNA encapsulation and reduce the toxicity associated with free PEI. These modifications improved the targeting specificity, cellular uptake, and therapeutic efficacy of the nano vectors for the delivery of miR17 to brain tumor cells (Fig. 3). In another study, grapefruit ELNs have undergone surface functionalization using a hydrophobic insertion technique to incorporate specific lipids, such as DSPE-PEG2000-Maleimide [19]. This modification process enables further changes through click chemistry, allowing the attachment of targeting ligands like the R11-3 aptamer (Fig. 3). The aptamer enhances cellular targeting by binding to specific receptors, including those on BBB endothelial cells, thereby improving uptake. The modified surface of grapefruit ELNs also exhibited alterations in zeta potential, confirming successful ligand attachment, while the PEG chains contribute to their biostability by reducing protein adsorption which further enhanced ELNs specificity, targeting, and therapeutic efficacy. In summary, plant-derived ELNs offer a promising nanoplatform for the treatment of neurological disorders. They exhibit high biocompatibility, effective targeting, and also the ability to deliver therapeutic biomacromolecules.

In addition, in the pursuit of improved glioma therapies, a study group have developed grapefruit-derived ELNs as a drug delivery system that integrates DOX-loaded heparin-based nanoparticles (DNs) with cRGD (cyclic Arginine-Glycine-Aspartic acid) for targeted therapy (Fig. 3) [131]. The DNs incorporate heparin to enhance drug loading capacity, achieving a four-fold increase compared to traditional methods. This allows for more efficient delivery of DOX, leading to improved anti-glioma efficacy. The DNs are designed with a pH-sensitive mechanism to facilitate the release of DOX in the acidic environment of tumor tissues, maximizing its therapeutic impact against glioma cells. cRGD is known to bind specifically to the integrin αvβ3 receptor, which is overexpressed in glioma cells. cRGD is conjugated with the carboxyl group of heparin in the DNs to improve targeting capabilities, allowing the ELNs to preferentially accumulate in glioma tissues via receptor-mediated endocytosis. Heparin-infused biomimetic delivery system enhances chemotherapy efficacy in brain tumors by stabilizing, prolonging circulation, and reducing immune response, improving survival rates and reducing pro-proliferative markers.

#### Drug Loading

ELNs derived from grapefruit represent a platform for neurotherapeutic drug development, providing a non-invasive and highly efficient approach to treating neurodegenerative disorders [133]. These toxic and nontoxic transporters can cross the BBB and deliver biomolecules directly to host cells, thereby preventing and alleviating immune suppression. These ELNs can be encapsulated in PEI to increase RNA and DNA encapsulation efficiency while reducing PEI toxicity. Systemic modifications, such as folic acid supplementation, can enhance targeting by activating donor-positive tumor cells. (Fig. 3). This receptor activation promotes the release of therapeutic miRNAs, such as miR17, which can downregulate major histocompatibility complex 1 (MHC1) expression, activate natural killer cells, and inhibit tumor growth. Intranasal administration of FA-coated grapefruit-derived ELNs ensures rapid and efficient delivery, reaching brain regions such as the olfactory bulb and hippocampus within hours, and also exhibits low toxicity and prolonged distribution [133]. The ability of grapefruit-derived ELNs to incorporate and protect biomolecules such as RNA shows its potential in gene therapy, especially for brain tumor treatment.

Grapefruit-derived ELNs can also be utilized for the encapsulation of therapeutic agents through physical techniques like probe sonication. This method enables the loading of drugs, including DOX, into the lipid bilayer structure of the vesicles (Fig. 3). The resulting loaded ELNs of grapefruit exhibit stability and can achieve controlled release of their cargo. Notably, these loaded vesicles demonstrate a pH-responsive release profile, where at physiological pH, approximately 45% of the drug is released within 48 h, while a more sustained release of up to 80% occurs over the same period under acidic conditions [19]. This pH-dependent release behavior ensures the efficient delivery of therapeutic agents to diseased areas, such as tumor microenvironments, while minimizing undesirable off-target effects.​

As one of innovative treatment strategies contributing to SCI repair, in a recent study, an anti-inflammatory and neuroprotective flavonoid called isoliquiritigenin (ISL) was loaded into ELNs derived from *Lycium barbarum L.* (indicated as ISL@PE), then incorporated into a 3D-printed bionic scaffold (Fig. 3) [110–112]. In the in vitro section, ISL contributed to the therapeutic potential of ELNs, where both created a favorable microenvironment for neuronal repair by modulating inflammation, decreasing ROS and pro-inflammatory cytokines IL-1β, IL-6, and TNF-α while upregulating IL-10, promoting the synaptic growth for neurons survival and enhancing the differentiation of neurons in NSCs, as evidenced by increased expression of markers like MAP2 and Tuj1. In addition, ISL@PE supported the phenotype transition M1-to-M2 when delivered to N9 microglial cells. iNOS and oxidative stress were reduced while anti-inflammatory (Arg-1, CD206) was upregulated. ISL@PE-incorporated 3D-printed hydrogels significantly improved motor function recovery, reduced glial scarring, and enhanced neuronal regeneration, with elevated levels of GAP43, NF200, and MBP indicating axonal growth and myelination in the rat model of SCI [110–112].

In the pursuit of innovative treatments for neuroinflammation, a recent study has demonstrated the drug carrying potential of ELNs derived from *Allium tuberosum*. A-ELNs were utilized as a novel drug delivery system to enhance the anti-inflammatory effects of dexamethasone in BV-2 and MG-6 cells derived from C57/BL6 mice [108]. Dexamethasone (Dex) was encapsulated within A-ELNs, forming Dex-A-ELNs, which demonstrated a significant increase in anti-inflammatory potency compared to either A-ELNs or Dex administered alone (Fig. 3). This synergistic effect was evidenced by a marked reduction in the levels of inflammatory cytokines and nitric oxide induced by LPS stimulation. Specifically, the treatment with Dex-A-ELNs resulted in a more substantial decrease in the expression of pro-inflammatory mediators such as iNOS and TNF-α, and IL-6, compared to the effects observed with A-ELNs or Dex alone. Furthermore, the mRNA expression of HO-1, was significantly elevated following treatment with Dex-A-ELNs, indicating an enhanced cellular response to oxidative stress and inflammation. The encapsulation of Dex within A-ELNs not only improved the translocation of the drug into both BV-2 and MG-6 cells but also altered the particle size, which may have contributed to improved pharmacokinetics and bioavailability.

#### Genetic engineering

Studies on transgenic plant-derived ELNs are unfortunately very limited. The only study conducted in this area is that a transgenic tomato was developed by a group to express crocin and picrocrocin genes from saffron; the biosynthetic genes responsible for these antioxidants were extracted from saffron and incorporated into the tomato genome via metabolic engineering (Fig. 3) [27]. This change sought to address the high cost and restricted availability of saffron by providing an efficient, scalable alternative for manufacturing these strong apocarotenoids. Tomafran, the bioengineered tomato, accumulates crocins and picrocrocin in its fruit, both of which have been linked to neuroprotection. Crocin-enriched Tomafran extracts, particularly when naturally encapsulated in purified Tomafran exosomes or loaded into chitosan nanoparticles, greatly increased neuronal survival in SH-SY5Y cells by reducing Okadaic acid-induced oxidative stress and tau hyperphosphorylation. This innovation demonstrates its promise as a low-cost treatment or nutraceutical for slowing AD development and improving brain function.

## Challenges and limitations

Although plant-derived ELNs present promising therapeutic potential, several limitations restrict their clinical translation for neurological disorder treatments [18]. A significant hindrance is their restricted ability to cross the blood–brain barrier (BBB) effectively, which is a crucial consideration in neurotherapeutics, particularly when contrasted with exosomes derived from mammalian sources that possess endogenous targeting capabilities [6, 7]. Furthermore, they typically lack inherent targeting specificity, necessitating post-isolation modifications like ligand conjugation or surface engineering to enhance cellular uptake and tissue selectivity [19]. The absence of uniform isolation and characterization methodologies further exacerbates batch-to-batch variations in yield, purity, and bioactive cargo, thereby affecting reproducibility across different research endeavors [47]. While plant-derived ELNs demonstrate enhanced biocompatibility and reduced immunogenicity compared to synthetic drug delivery systems, such as liposomes and polymeric nanoparticles, they generally exhibit diminished stability and less controlled release kinetics [35]. Moreover, their biological activity may differ depending on plant species, growth conditions, and extraction methods. The limited availability of in vivo neuropharmacological data, coupled with inadequate comprehensive safety assessments, raises concerns regarding their scalability and clinical applicability, underscoring the necessity for more thorough investigations in subsequent studies.

## Conclusion

Plant-derived ELNs represent a novel and promising drug delivery system for the treatment of neurological disorders, combining biotechnology and pharmaceutical approaches. Due to their biocompatibility, minimal toxicity, and ability to traverse the BBB, ELNs provide a targeted, non-invasive approach for addressing neurological disorders. Specifically, surface modifications and loading with therapeutic molecules that improve therapeutic efficacy present prospects for the development of novel treatment techniques for neurodegenerative diseases and other central nervous system disorders.

Nonetheless, it is crucial to acknowledge that the incorporation of ELNs into clinical applications remains at its very early stages. Further experimental and clinical investigations are required to assess their efficacy, safety, dosage optimization, and long-term biological impacts. Future investigations will further the understanding of the pharmacokinetic features of plant-derived exosomes, their impact on immunological responses, and their therapeutic efficacy in diverse neurological disorders. At present, the majority of research on plant-derived ELNs is in experimental and preclinical stages, with clinical trials yet to begin.

In summary, plant-derived ELNs possess revolutionary potential in the management of neurological disorders and may provide effective therapeutic alternatives in the future. Nonetheless, for this technology to go to clinical use, extensive human research and scientific proof are necessary.

## Data Availability

The datasets generated during and/or analysed during the current study are available from the corresponding author on reasonable request.

## References

[1] Janas AM, Sapoń K, Janas T, Stowell MHB, Janas T. Exosomes and other extracellular vesicles in neural cells and neurodegenerative diseases. Biochimica et Biophysica Acta (BBA) - Biomembranes. 2016;1858(6):1139–51. 10.1016/j.bbamem.2016.02.011. 26874206 10.1016/j.bbamem.2016.02.011

[2] Dad HA, Gu T-W, Zhu A-Q, Huang L-Q, Peng L-H. Plant exosome-like nanovesicles: emerging therapeutics and drug delivery nanoplatforms. Mol Ther. 2021;29(1):13–31. 10.1016/j.ymthe.2020.11.030. 33278566 10.1016/j.ymthe.2020.11.030 PMC7791080

[3] Subha D, Harshnii K, Madhikiruba KG, Nandhini M, Tamilselvi KS. Plant derived exosome- like nanovesicles: an updated overview. Plant Nano Biology. 2023. 10.1016/j.plana.2022.100022.

[4] Mu J, Zhuang X, Wang Q, Jiang H, Deng Z, Wang B, Zhang L, Kakar S, Jun Y, Miller D, Zhang H. Interspecies communication between plant and mouse gut host cells through edible plant derived exosome-like nanoparticles. Mol Nutr Food Res. 2014;58(7):1561–73. 10.1002/mnfr.201300729. 24842810 10.1002/mnfr.201300729 PMC4851829

[5] Berridge MJ, Bootman MD, Roderick HL. Calcium signalling: dynamics, homeostasis and remodelling. Nat Rev Mol Cell Biol. 2003;4(7):517–29. 10.1038/nrm1155. 12838335 10.1038/nrm1155

[6] Wang R, Zhang Y, Guo Y, Zeng W, Li J, Wu J, Li N, Zhu A, Li J, Di L, Cao P. Plant-derived nanovesicles: promising therapeutics and drug delivery nanoplatforms for brain disorders. Fundam Res. 2023. 10.1016/j.fmre.2023.09.007. 40242551 10.1016/j.fmre.2023.09.007 PMC11997602

[7] Wang Y, Sun SK, Liu Y, Zhang Z. Advanced hitchhiking nanomaterials for biomedical applications. Theranostics. 2023;13(14):4781–801. 10.7150/THNO.88002. 37771786 10.7150/thno.88002 PMC10526662

[8] Gong B, Radulovic M, Figueiredo-Pereira ME, Cardozo C. The Ubiquitin-Proteasome System: Potential Therapeutic Targets for Alzheimer’s Disease and Spinal Cord Injury. Front Mol Neurosci.e 2016;9. 10.3389/fnmol.2016.00004 10.3389/fnmol.2016.00004 PMC4727241 26858599

[9] Isik S, Yeman Kiyak B, Akbayir R, Seyhali R, Arpaci T. Microglia Mediated Neuroinflammation in Parkinson’s Disease. In: Cells (Vol. 12, Issue 7); 2023. MDPI. 10.3390/cells12071012. 10.3390/cells12071012 PMC10093562 37048085

[10] Khan J, Rudrapal M, Bhat EA, Ali A, Alaidarous M, Alshehri B, Banwas S, Ismail R, Egbuna C. Perspective insights to bio-nanomaterials for the treatment of neurological disorders. Front Bioeng Biotechnol. 2021. 10.3389/fbioe.2021.724158. 34712651 10.3389/fbioe.2021.724158 PMC8546296

[11] Zhang M, Xiang C, Niu R, He X, Luo W, Liu W, Gu R. Liposomes as versatile agents for the management of traumatic and nontraumatic central nervous system disorders: drug stability, targeting efficiency, and safety. Neural Regen Res. 2025;20(7):1883–99. 10.4103/NRR.NRR-D-24-00048. 39254548 10.4103/NRR.NRR-D-24-00048 PMC11691476

[12] Ruffo P, De Amicis F, Giardina E, Conforti F. Long-noncoding RNAs as epigenetic regulators in neurodegenerative diseases. Neural Regen Res. 2023;18(6):1243. 10.4103/1673-5374.358615. 36453400 10.4103/1673-5374.358615 PMC9838156

[13] Zhou S, Yu X, Wang M, Meng Y, Song D, Yang H, Wang D, Bi J, Xu S. Long non-coding RNAs in pathogenesis of neurodegenerative diseases. Front Cell Dev Biol. 2021. 10.3389/fcell.2021.719247. 34527672 10.3389/fcell.2021.719247 PMC8435612

[14] Kim SQ, Kim KH. Emergence of edible plant-derived nanovesicles as functional food components and nanocarriers for therapeutics delivery: potentials in human health and disease. Cells. 2022. 10.3390/cells11142232. 35883674 10.3390/cells11142232 PMC9319657

[15] Manzaneque-López MC, Sánchez-López CM, Pérez-Bermúdez P, Soler C, Marcilla A. Dietary-derived exosome-like nanoparticles as bacterial modulators: beyond micrornas. Nutrients. 2023;15(5): 1265. 10.3390/nu15051265. 36904264 10.3390/nu15051265 PMC10005434

[16] Chen X, Zhou Y, Yu J. Exosome-like nanoparticles from Ginger rhizomes inhibited NLRP3 inflammasome activation. Mol Pharm. 2019;16(6):2690–9. 10.1021/acs.molpharmaceut.9b00246. 31038962 10.1021/acs.molpharmaceut.9b00246

[17] Zhang L, He F, Gao L, Cong M, Sun J, Xu J, Wang Y, Hu Y, Asghar S, Hu L, Qiao H. Engineering exosome-like nanovesicles derived from *Asparagus cochinchinensis* can inhibit the proliferation of hepatocellular carcinoma cells with better safety profile. Int J Nanomedicine. 2021;16:1575–86. 10.2147/IJN.S293067. 33664572 10.2147/IJN.S293067 PMC7924256

[18] Li Y, Wang Y, Zhao H, Pan Q, Chen G. Engineering strategies of plant-derived exosome-like nanovesicles: current knowledge and future perspectives. Int J Nanomedicine. 2024;19:12793–815. 10.2147/IJN.S496664. 39640047 10.2147/IJN.S496664 PMC11618857

[19] Moon K, Hur J, Kim KP, Lee K, Kang JY. Surface-functionalizable plant-derived extracellular vesicles for targeted drug delivery carrier using grapefruit. Adv Mater Interfaces. 2023;10(22): 2300220. 10.1002/ADMI.202300220.

[20] Woith E, Guerriero G, Hausman JF, Renaut J, Leclercq CC, Weise C, Legay S, Weng A, Melzig MF. Plant extracellular vesicles and nanovesicles: focus on secondary metabolites, proteins and lipids with perspectives on their potential and sources. Int J Mol Sci. 2021. 10.3390/ijms22073719. 33918442 10.3390/ijms22073719 PMC8038311

[21] Zhang Y, Liu Y, Liu H, Tang WH. Exosomes: biogenesis, biologic function and clinical potential. Cell Biosci. 2019;9(1): 19. 10.1186/s13578-019-0282-2. 30815248 10.1186/s13578-019-0282-2 PMC6377728

[22] Wang J, Ding Y, Wang J, Hillmer S, Miao Y, Lo SW, Wang X, Robinson DG, Jiang L. EXPO, an exocyst-positive organelle distinct from multivesicular endosomes and autophagosomes, mediates cytosol to cell wall exocytosis in arabidopsis and tobacco cells. Plant Cell. 2010;22(12):4009–30. 10.1105/tpc.110.080697. 21193573 10.1105/tpc.110.080697 PMC3027174

[23] Gioia SD, Hossain MN, Conese M. Biological properties and therapeutic effects of plant-derived nanovesicles. Open Med. 2020;15(1):1096–122. 10.1515/MED-2020-0160. 10.1515/med-2020-0160 PMC7718644 33336066

[24] Tang C-H, Huang T-H, Chang C-S, Fu W-M, Yang R-S. Water solution of onion crude powder inhibits RANKL-induced osteoclastogenesis through ERK, p38 and NF-κB pathways. Osteoporos Int. 2009;20(1):93–103. 10.1007/s00198-008-0630-2. 18506384 10.1007/s00198-008-0630-2

[25] Woith E, Melzig M. Extracellular vesicles from fresh and dried plants—simultaneous purification and visualization using gel electrophoresis. Int J Mol Sci. 2019;20(2): 357. 10.3390/ijms20020357. 30654488 10.3390/ijms20020357 PMC6359398

[26] Fymat AL. Surgical and Non-Surgical Management and Treatment of Glioblastoma: I. Primary Tumors. Open Access J Surg. 2017;7(2). 10.19080/OAJS.2017.07.555706.

[27] Garuffi M, Costa JLR, Hernández SSS, Vital TM, Stein AM, Santos JGdos, Stella F. Effects of resistance training on the performance of activities of daily living in patients with Alzheimer’s disease. Geriatr Gerontol Int. 2013;13(2):322–8. 10.1111/j.1447-0594.2012.00899.x. 22726761 10.1111/j.1447-0594.2012.00899.x

[28] Dolma L, Damodaran A, Panonnummal R, Nair SC. Exosomes isolated from citrus lemon: a promising candidate for the treatment of Alzheimer’s disease. Ther Deliv. 2024;15(7):507–19. 10.1080/20415990.2024.2354119. 38888652 10.1080/20415990.2024.2354119 PMC11412142

[29] Pocsfalvi G, Turiák L, Ambrosone A, del Gaudio P, Puska G, Fiume I, Silvestre T, Vékey K. Protein biocargo of citrus fruit-derived vesicles reveals heterogeneous transport and extracellular vesicle populations. J Plant Physiol. 2018;229:111–21. 10.1016/j.jplph.2018.07.006. 30056374 10.1016/j.jplph.2018.07.006

[30] Yu T, Xu Y, Ahmad MA, Javed R, Hagiwara H, Tian X. Exosomes as a promising therapeutic strategy for peripheral nerve injury. Curr Neuropharmacol. 2021;19(12):2141–51. 10.2174/1570159X19666210203161559. 33535957 10.2174/1570159X19666210203161559 PMC9185764

[31] Pinedo M, de la Canal L, de Marcos Lousa C. A call for rigor and standardization in plant extracellular vesicle research. J Extracell Vesicles. 2021. 10.1002/jev2.12048. 33936567 10.1002/jev2.12048 PMC8077130

[32] Jones-Rhoades MW, & Bartel DP (2004) Computational Identification of Plant MicroRNAs and Their Targets, Including a Stress-Induced miRNA The primary method of identifying miRNA genes has been to isolate, reverse transcribe, clone, and sequence small cellular RNAs (Lagos-Quintana et al., 2001; Lau et. In *Molecular Cell* (Vol. 14). Lee and Ambros. http://www.molecule. 10.1016/j.molcel.2004.05.027 15200956

[33] Vaucheret H, Chupeau Y. Ingested plant miRNAs regulate gene expression in animals. Cell Res. 2012;22(1):3–5. 10.1038/cr.2011.164. 22025251 10.1038/cr.2011.164 PMC3351922

[34] Liu YC, Chen WL, Kung WH, Huang HD. Plant miRNAs found in human circulating system provide evidences of cross kingdom RNAi. BMC Genomics. 2017. 10.1186/s12864-017-3502-3. 28361700 10.1186/s12864-017-3502-3 PMC5374554

[35] Tinnirello V, Rabienezhad Ganji N, De Marcos Lousa C, Alessandro R, Raimondo S. Exploiting the opportunity to use plant-derived nanoparticles as delivery vehicles. Plants. 2023;12(6):1207. 10.3390/PLANTS12061207. 36986896 10.3390/plants12061207 PMC10053153

[36] Yu L, Deng Z, Liu L, Zhang W, Wang C. Plant-derived nanovesicles: a novel form of nanomedicine. Front Bioeng Biotechnol. 2020;8: 584391. 10.3389/FBIOE.2020.584391. 33154966 10.3389/fbioe.2020.584391 PMC7591720

[37] Onelli E, Prescianotto-Baschong C, Caccianiga M, Moscatelli A. Clathrin-dependent and independent endocytic pathways in tobacco protoplasts revealed by labelling with charged nanogold. J Exp Bot. 2008;59(11):3051–68. 10.1093/JXB/ERN154. 18603619 10.1093/jxb/ern154 PMC2504345

[38] Chen X, Irani NG, Friml J. Clathrin-mediated endocytosis: the gateway into plant cells. Curr Opin Plant Biol. 2011;14(6):674–82. 10.1016/J.PBI.2011.08.006. 21945181 10.1016/j.pbi.2011.08.006

[39] Kou L, Sun J, Zhai Y, He Z. The endocytosis and intracellular fate of nanomedicines: implication for rational design. Asian J Pharm Sci. 2013;8(1):1–10. 10.1016/J.AJPS.2013.07.001.

[40] Jauch EC, Saver JL, Adams HP, Bruno A, Connors JJ, Demaerschalk BM, Khatri P, McMullan PW, Qureshi AI, Rosenfield K, Scott PA, Summers DR, Wang DZ, Wintermark M, Yonas H. Guidelines for the early management of patients with acute ischemic stroke. Stroke. 2013;44(3):870–947. 10.1161/STR.0b013e318284056a. 23370205 10.1161/STR.0b013e318284056a

[41] Ly NP, Han HS, Kim M, Park JH, Choi KY. Plant-derived nanovesicles: current understanding and applications for cancer therapy. Bioact Mater. 2023;22:365–83. 10.1016/J.BIOACTMAT.2022.10.005. 36311046 10.1016/j.bioactmat.2022.10.005 PMC9588993

[42] Oh N, Park JH. Endocytosis and exocytosis of nanoparticles in mammalian cells. Int J Nanomedicine. 2014;9(Supplement 1):51–63. 10.2147/IJN.S26592. 24872703 10.2147/IJN.S26592 PMC4024976

[43] Tan ZL, Li JF, Luo HM, Liu YY, Jin Y. Plant extracellular vesicles: a novel bioactive nanoparticle for tumor therapy. Front Pharmacol. 2022;13: 1006299. 10.3389/FPHAR.2022.1006299. 36249740 10.3389/fphar.2022.1006299 PMC9559701

[44] Li A, Li D, Gu Y, Liu R, Tang X, Zhao Y, Qi F, Wei J, Liu J. Plant-derived nanovesicles: further exploration of biomedical function and application potential. Acta Pharm Sin B. 2023;13(8):3300–20. 10.1016/J.APSB.2022.12.022. 37655320 10.1016/j.apsb.2022.12.022 PMC10465964

[45] Li D, Tang Q, Yang M, Xu H, Zhu M, Zhang Y, Tian C, Nie Y, Wang J, Liang Y, Wang L, Yao J. Plant-derived exosomal nanoparticles: potential therapeutic for inflammatory bowel disease. Nanoscale Adv. 2023;5(14):3575–88. 10.1039/D3NA00093A. 37441251 10.1039/d3na00093a PMC10334410

[46] Li S, Zhang R, Wang A, Li Y, Zhang M, Kim J, Zhu Y, Wang Q, Zhang Y, Wei Y, Wang J. *Panax notoginseng*: derived exosome-like nanoparticles attenuate ischemia reperfusion injury via altering microglia polarization. J Nanobiotechnology. 2023;21(1): 416. 10.1186/s12951-023-02161-1. 37946257 10.1186/s12951-023-02161-1 PMC10636993

[47] Xu Z, Xu Y, Zhang K, Liu Y, Liang Q, Thakur A, Liu W, Yan Y. Plant-derived extracellular vesicles (PDEVs) in nanomedicine for human disease and therapeutic modalities. J Nanobiotechnology. 2023;21(1):1–13. 10.1186/S12951-023-01858-7. 36978093 10.1186/s12951-023-01858-7 PMC10049910

[48] Blanco E, Shen H, Ferrari M. Principles of nanoparticle design for overcoming biological barriers to drug delivery. Nat Biotechnol. 2015;33(9):941–51. 10.1038/nbt.3330. 26348965 10.1038/nbt.3330 PMC4978509

[49] Marsh M. Clathrin-coated vesicles and receptor-mediated endocytosis. Encycl Life Sci. 2001. 10.1038/NPG.ELS.0000555.

[50] Zacheo A, Bizzarro L, Blasi L, Piccirillo C, Cardone A, Gigli G, Ragusa A, Quarta A. Lipid-based nanovesicles for simultaneous intracellular delivery of hydrophobic, hydrophilic, and amphiphilic species. Frontiers in Bioengineering and Biotechnology. 2020;8:547416. 10.3389/FBIOE.2020.00690/BIBTEX. 10.3389/fbioe.2020.00690 PMC7350901 32719782

[51] Leggio L, Paternò G, Vivarelli S, L’episcopo F, Tirolo C, Raciti G, Pappalardo F, Giachino C, Caniglia S, Serapide MF, Marchetti B, Iraci N. Extracellular vesicles as nanotherapeutics for Parkinson’s disease. Biomolecules. 2020;10(9): 1327. 10.3390/BIOM10091327. 32948090 10.3390/biom10091327 PMC7563168

[52] Tönnies E, Trushina E. Oxidative stress, synaptic dysfunction, and Alzheimer’s disease. J Alzheimers Dis. 2017;57(4):1105–21. 10.3233/JAD-161088. 28059794 10.3233/JAD-161088 PMC5409043

[53] Hampel H, Hardy J, Blennow K, Chen C, Perry G, Kim SH, Villemagne VL, Aisen P, Vendruscolo M, Iwatsubo T, Masters CL, Cho M, Lannfelt L, Cummings JL, Vergallo A. The amyloid-β pathway in Alzheimer’s disease. Mol Psychiatry. 2021;26(10):5481–503. 10.1038/s41380-021-01249-0. 34456336 10.1038/s41380-021-01249-0 PMC8758495

[54] Kinney JW, Bemiller SM, Murtishaw AS, Leisgang AM, Salazar AM, Lamb BT. Inflammation as a central mechanism in Alzheimer’s disease. Alzheimers Dement (N Y). 2018;4(1):575–90. 10.1016/j.trci.2018.06.014. 30406177 10.1016/j.trci.2018.06.014 PMC6214864

[55] Uzbekov MG. Monoamine oxidase as a potential biomarker of the efficacy of treatment of mental disorders. Biochem Mosc. 2021;86(6):773–83. 10.1134/S0006297921060146. 10.1134/S0006297921060146 34225599

[56] Wang R, Reddy PH. Role of glutamate and NMDA receptors in Alzheimer’s disease. J Alzheimers Dis. 2017;57(4):1041–8. 10.3233/JAD-160763. 27662322 10.3233/JAD-160763 PMC5791143

[57] Zuo L, Hemmelgarn BT, Chuang C-C, Best TM. The role of oxidative stress-induced epigenetic alterations in amyloid- *β* production in Alzheimer’s disease. Oxid Med Cell Longev. 2015;2015:1–13. 10.1155/2015/604658. 10.1155/2015/604658 PMC4620382 26543520

[58] Liu B, Hong J-S. Role of microglia in inflammation-mediated neurodegenerative diseases: mechanisms and strategies for therapeutic intervention. J Pharmacol Exp Ther. 2003;304(1):1–7. 10.1124/jpet.102.035048. 12490568 10.1124/jpet.102.035048

[59] Švob Štrac D, Pivac N, Mück-Šeler D. The serotonergic system and cognitive function. Transl Neurosci. 2016;7(1):35–49. 10.1515/tnsci-2016-0007. 28123820 10.1515/tnsci-2016-0007 PMC5017596

[60] Martin F. A clinical overview of cholinesterase inhibitors in Alzheimer’s disease. Int Psychogeriatr. 2002;14:93–126. 10.1017/S1041610203008688. 12636182 10.1017/s1041610203008688

[61] Chowdhury S. Monoclonal antibody treatments for Alzheimer’s disease: aducanumab and lecanemab. Discoveries (Craiova). 2023;11(3):e173. 10.15190/D.2023.12. 39726673 10.15190/d.2023.12 PMC11671221

[62] Sevigny J, Chiao P, Bussière T, Weinreb PH, Williams L, Maier M, Dunstan R, Salloway S, Chen T, Ling Y, O’Gorman J, Qian F, Arastu M, Li M, Chollate S, Brennan MS, Quintero-Monzon O, Scannevin RH, Arnold HM, Engber T, Rhodes K, Ferrero J, Hang Y, Mikulskis A, Grimm J, Hock C, Nitsch RM, Sandrock A. Addendum: the antibody aducanumab reduces Aβ plaques in Alzheimer’s disease. Nature. 2017;546(7659):564–564. 10.1038/nature22809. 28640269 10.1038/nature22809

[63] Manfredsson FP, Luk KC, Benskey MJ, Gezer A, Garcia J, Kuhn NC, Sandoval IM, Patterson JR, O’Mara A, Yonkers R, Kordower JH. Induction of alpha-synuclein pathology in the enteric nervous system of the rat and non-human primate results in gastrointestinal dysmotility and transient CNS pathology. Neurobiol Dis. 2018;112:106–18. 10.1016/J.NBD.2018.01.008. 29341898 10.1016/j.nbd.2018.01.008 PMC5890443

[64] Scarmeas N, Stern Y, Tang M, Mayeux R, Luchsinger JA. Mediterranean diet and risk for Alzheimer’s disease. Ann Neurol. 2006;59(6):912–21. 10.1002/ana.20854. 16622828 10.1002/ana.20854 PMC3024594

[65] Sitzer DI, Twamley EW, Jeste DV. Cognitive training in Alzheimer’s disease: a meta-analysis of the literature. Acta Psychiatr Scand. 2006;114(2):75–90. 10.1111/J.1600-0447.2006.00789.X. 16836595 10.1111/j.1600-0447.2006.00789.x

[66] Moustafa AA, Chakravarthy S, Phillips JR, Gupta A, Keri S, Polner B, Frank MJ, Jahanshahi M. Motor symptoms in Parkinson’s disease: a unified framework. Neurosci Biobehav Rev. 2016;68:727–40. 10.1016/j.neubiorev.2016.07.010. 27422450 10.1016/j.neubiorev.2016.07.010

[67] Schapira AHV, Chaudhuri KR, Jenner P. Non-motor features of Parkinson disease. Nat Rev Neurosci. 2017;18(7):435–50. 10.1038/nrn.2017.62. 28592904 10.1038/nrn.2017.62

[68] Mattson MP. Apoptosis in neurodegenerative disorders. Nat Rev Mol Cell Biol. 2000;1(2):120–30. 10.1038/35040009. 11253364 10.1038/35040009

[69] Srinivasan E, Chandrasekhar G, Chandrasekar P, Anbarasu K, Vickram AS, Karunakaran R, Rajasekaran R, Srikumar PS. Alpha-synuclein aggregation in Parkinson’s disease. Front Med. 2021. 10.3389/fmed.2021.736978. 10.3389/fmed.2021.736978 PMC8558257 34733860

[70] Mesfin FB, Karsonovich T, Al-Dhahir MA. Gliomas. StatPearls. 2024. https://www.ncbi.nlm.nih.gov/books/NBK441874/. Accessed 6 Oct 2024. 28722904

[71] Stefanis L. Synuclein in Parkinson’s disease. Cold Spring Harb Perspect Med. 2012;2(2): a009399. 10.1101/cshperspect.a009399. 22355802 10.1101/cshperspect.a009399 PMC3281589

[72] Etxebeste-Mitxeltorena M, Niza E, Fajardo CM, Gil C, Gómez-Gómez L, Martinez A, Ahrazem O. Neuroprotective properties of exosomes and chitosan nanoparticles of Tomafran, a bioengineered tomato enriched in crocins. Nat Prod Bioprospect. 2024. 10.1007/s13659-023-00425-9. 38212507 10.1007/s13659-023-00425-9 PMC10784249

[73] Huttner A. Overview of primary brain tumors: pathologic classification, epidemiology, molecular biology, and prognostic markers. Hematol Oncol Clin North Am. 2012;26(4):715–32. 10.1016/J.HOC.2012.05.004. 22794280 10.1016/j.hoc.2012.05.004

[74] Mulcahy EQX, Colón RR, Abounader R. HGF/MET signaling in malignant brain tumors. International Journal of Molecular Sciences. 2020;21(20):7546. 10.3390/IJMS21207546. 33066121 10.3390/ijms21207546 PMC7590206

[75] Murakami M, Tognini P. The circadian clock as an essential molecular link between host physiology and microorganisms. Front Cell Infect Microbiol. 2020;9: 500180. 10.3389/FCIMB.2019.00469. 10.3389/fcimb.2019.00469 PMC6987142 32039048

[76] Strong MJ, Garces J, Carlos Vera J, Mathkour M, Emerson N, Ware ML. Brain Tumors: Epidemiology and Current Trends in Treatment. 2016. 10.4172/2475-3203.1000102.

[77] Szklener K, Mazurek M, Wieteska M, Wacławska M, Bilski M, Mańdziuk S. New Directions in the Therapy of Glioblastoma. Cancers. 2022;14:5377. 10.3390/CANCERS14215377. 10.3390/cancers14215377 PMC9655599 36358795

[78] Obrenovich MEM. Leaky gut, leaky brain? Microorganisms. 2018;6(4):107. 10.3390/MICROORGANISMS6040107. 30340384 10.3390/microorganisms6040107 PMC6313445

[79] Delhalle S, Bode SFN, Balling R, Ollert M, He FQ. A roadmap towards personalized immunology. Npj Syst Biol Appl. 2018;4(1):1–14. 10.1038/s41540-017-0045-9. 29423275 10.1038/s41540-017-0045-9 PMC5802799

[80] Rick JW, Shahin M, Chandra A, Dalle Ore C, Yue JK, Nguyen A, Yagnik G, Sagar S, Arfaie S, Aghi MK. Systemic therapy for brain metastases. Crit Rev Oncol Hematol. 2019;142:44–50. 10.1016/J.CRITREVONC.2019.07.012. 31357143 10.1016/j.critrevonc.2019.07.012 PMC6746616

[81] Parrish KE, Sarkaria JN, Elmquist WF. Improving drug delivery to primary and metastatic brain tumors: strategies to overcome the blood–brain barrier. Clin Pharmacol Ther. 2015;97(4):336–46. 10.1002/CPT.71. 25669487 10.1002/cpt.71

[82] Haumann R, Videira JC, Kaspers GJL, van Vuurden DG, Hulleman E. Overview of current drug delivery methods across the blood-brain barrier for the treatment of primary brain tumors. CNS Drugs. 2020;34(11):1121–31. 10.1007/S40263-020-00766-W. 32965590 10.1007/s40263-020-00766-w PMC7658069

[83] Li Y, & Yang G-Y. Pathophysiology of Ischemic Stroke. Transl Res Stroke*.* 1st ed. 2017. pp. 51–75. 10.1007/978-981-10-5804-2_4.

[84] Musuka TD, Wilton SB, Traboulsi M, Hill MD. Diagnosis and management of acute ischemic stroke: speed is critical. Can Med Assoc J. 2015;187(12):887–93. 10.1503/cmaj.140355. 26243819 10.1503/cmaj.140355 PMC4562827

[85] Dm S, Cohen Phd G, Karolinska, Wardlaw JM, Murray V, Berge E, Del Zoppo G, Sandercock P, Lindley RL, Cohen G (2012) Articles Recombinant tissue plasminogen activator for acute ischaemic stroke: an updated systematic review and meta-analysis. The Lancet 379:2364–2372. 10.1016/S0140 10.1016/S0140-6736(12)60738-7 PMC3386494 22632907

[86] Bareford LM, Swaan PW. Endocytic mechanisms for targeted drug delivery. Adv Drug Deliv Rev. 2007;59(8):748–58. 10.1016/J.ADDR.2007.06.008. 17659804 10.1016/j.addr.2007.06.008 PMC2000329

[87] Anjum A, Yazid MD, Fauzi Daud M, Idris J, Ng AMH, Selvi Naicker A, Ismail OHR, Athi Kumar RK, Lokanathan Y. Spinal cord injury: pathophysiology, multimolecular interactions, and underlying recovery mechanisms. Int J Mol Sci. 2020;21(20): 7533. 10.3390/ijms21207533. 33066029 10.3390/ijms21207533 PMC7589539

[88] Ham PB, Raju R. Mitochondrial function in hypoxic ischemic injury and influence of aging. Prog Neurobiol. 2017;157:92–116. 10.1016/j.pneurobio.2016.06.006. 27321753 10.1016/j.pneurobio.2016.06.006 PMC5161736

[89] Sittipo P, Choi J, Lee S, Lee YK. The function of gut microbiota in immune-related neurological disorders: a review. J Neuroinflammation. 2022;19(1):1–17. 10.1186/S12974-022-02510-1. 35706008 10.1186/s12974-022-02510-1 PMC9199126

[90] Brodell DW, Jain A, Elfar JC, Mesfin A. National trends in the management of central cord syndrome: an analysis of 16,134 patients. Spine J. 2015;15(3):435–42. 10.1016/j.spinee.2014.09.015. 25264321 10.1016/j.spinee.2014.09.015 PMC4339306

[91] Forman HJ, Zhang H. Author correction: targeting oxidative stress in disease: promise and limitations of antioxidant therapy. Nat Rev Drug Discov. 2021;20(8):652–652. 10.1038/s41573-021-00267-5. 34257433 10.1038/s41573-021-00267-5 PMC8414784

[92] Fehlings MG, Vaccaro A, Wilson JR, Singh A, Cadotte WD, Harrop JS, Aarabi B, Shaffrey C, Dvorak M, Fisher C, Arnold P, Massicotte EM, Lewis S, Rampersaud R. Early versus delayed decompression for traumatic cervical spinal cord injury: results of the Surgical Timing in Acute Spinal Cord Injury Study (STASCIS). PLoS One. 2012;7(2):e32037. 10.1371/journal.pone.0032037. 22384132 10.1371/journal.pone.0032037 PMC3285644

[93] Kang L, Zhang H, Jia C, Zhang R, Shen C. Targeting oxidative stress and inflammation in intervertebral disc degeneration: therapeutic perspectives of phytochemicals. Front Pharmacol. 2022. 10.3389/fphar.2022.956355. 35903342 10.3389/fphar.2022.956355 PMC9315394

[94] Liu L, Huh JR, Shah K. Microbiota and the gut-brain-axis: implications for new therapeutic design in the CNS. EBioMedicine. 2022;77: 103908. 10.1016/J.EBIOM.2022.103908. 35255456 10.1016/j.ebiom.2022.103908 PMC8897630

[95] Sundaram K, Mu J, Kumar A, Behera J, Lei C, Sriwastva MK, Xu F, Dryden GW, Zhang L, Chen S, Yan J, Zhang X, Park JW, Merchant ML, Tyagi N, Teng Y, Zhang H-G. Garlic exosome-like nanoparticles reverse high-fat diet induced obesity via the gut/brain axis. Theranostics. 2022;12(3):1220–46. 10.7150/thno.65427. 35154484 10.7150/thno.65427 PMC8771565

[96] Weltens N, Iven J, Van Oudenhove L, Kano M. The gut–brain axis in health neuroscience: implications for functional gastrointestinal disorders and appetite regulation. Ann N Y Acad Sci. 2018;1428(1):129–50. 10.1111/NYAS.13969. 30255954 10.1111/nyas.13969

[97] Szala-Rycaj J, Szewczyk A, Zagaja M, Kaczmarczyk-Ziemba A, Maj M, Andres-Mach M. The influence of topinambur and inulin preventive supplementation on microbiota, anxious behavior, cognitive functions and neurogenesis in mice exposed to the chronic unpredictable mild stress. Nutrients. 2023;15(9): 2041. 10.3390/NU15092041. 37432210 10.3390/nu15092041 PMC10181297

[98] Fock E, Parnova R. Mechanisms of blood-brain barrier protection by microbiota-derived short-chain fatty acids. Cells. 2023;12(4):657. 10.3390/CELLS12040657. 36831324 10.3390/cells12040657 PMC9954192

[99] Wekerle H. Brain autoimmunity and intestinal microbiota: 100 trillion game changers. Trends Immunol. 2017;38(7):483–97. 10.1016/J.IT.2017.03.008. 28601415 10.1016/j.it.2017.03.008

[100] Mayer EA, Padua D, Tillisch K. Altered brain-gut axis in autism: comorbidity or causative mechanisms? Bioessays. 2014;36(10):933–9. 10.1002/BIES.201400075. 25145752 10.1002/bies.201400075

[101] Hey G, Nair N, Klann E, Gurrala A, Safarpour D, Mai V, Ramirez-Zamora A, Vedam-Mai V. Therapies for Parkinson’s disease and the gut microbiome: evidence for bidirectional connection. Front Aging Neurosci. 2023;15: 1151850. 10.3389/FNAGI.2023.1151850. 37323145 10.3389/fnagi.2023.1151850 PMC10261989

[102] Lazaro T, Brastianos PK. Immunotherapy and targeted therapy in brain metastases: emerging options in precision medicine. CNS Oncol. 2017;6(2):139–51. 10.2217/CNS-2016-0038. 28425754 10.2217/cns-2016-0038 PMC6020878

[103] Zhu X, Han Y, Du J, Liu R, Jin K, Yi W, Zhu X, Han Y, Du J, Liu R, Jin K, Yi W. Microbiota-gut-brain axis and the central nervous system. Oncotarget. 2017;8(32):53829–38. 10.18632/ONCOTARGET.17754. 28881854 10.18632/oncotarget.17754 PMC5581153

[104] Ullah H, Arbab S, Tian Y, Liu CQ, Chen Y, Qijie L, Khan MIU, Hassan IU, Li K. The gut microbiota–brain axis in neurological disorder. Front Neurosci. 2023;17: 1225875. 10.3389/FNINS.2023.1225875. 37600019 10.3389/fnins.2023.1225875 PMC10436500

[105] Daban C, Vieta E, Mackin P, Young AH. Hypothalamic-pituitary-adrenal axis and bipolar disorder. Psychiatr Clin North Am. 2005;28(2):469–80. 10.1016/J.PSC.2005.01.005. 15826743 10.1016/j.psc.2005.01.005

[106] Vestuto V, Conte M, Vietri M, Mensitieri F, Santoro V, Di Muro A, Alfieri M, Moros M, Miranda MR, Amante C, Delli Carri M, Campiglia P, Dal Piaz F, Del Gaudio P, De Tommasi N, Leone A, Moltedo O, Pepe G, Cappetta E, Ambrosone A. Multiomic profiling and neuroprotective bioactivity of Salvia hairy root-derived extracellular vesicles in a cellular model of Parkinson’s disease. Int J Nanomed. 2024;19:9373–93. 10.2147/IJN.S479959. 10.2147/IJN.S479959 PMC11403015 39286353

[107] Wang B, Guo X-J, Cai H, Zhu Y-H, Huang L-Y, Wang W, Luo L, Qi S-H. *Momordica charantia*-derived extracellular vesicles-like nanovesicles inhibited glioma proliferation, migration, and invasion by regulating the PI3K/AKT signaling pathway. J Funct Foods. 2022;90: 104968. 10.1016/j.jff.2022.104968.

[108] Wang D-K, Guan L, Li D-Y, Zhao B-F, Li Z-P, Liu Y, Bai M-Y, Lu Y-J, Shen Z-L, Zhou Z-P, Zhang C-J, Mei X-F. Onion-derived nanoparticles ameliorate the microenvironment to revitalize motor function after spinal cord injury. ACS Appl Nano Mater. 2024;7(19):22694–713. 10.1021/acsanm.4c03645.

[109] Kawada K, Ishida T, Morisawa S, Jobu K, Higashi Y, Aizawa F, Yagi K, Izawa-Ishizawa Y, Niimura T, Abe S, Goda M, Miyamura M, Ishizawa K. *Atractylodes lancea* (Thunb.) DC. [Asteraceae] rhizome-derived exosome-like nanoparticles suppress lipopolysaccharide-induced inflammation in murine microglial cells. Front Pharmacol. 2024. 10.3389/fphar.2024.1302055. 38738173 10.3389/fphar.2024.1302055 PMC11082290

[110] Wang Q, Liu K, Cao X, Rong W, Shi W, Yu Q, Deng W, Yu J, Xu X. Plant-derived exosomes extracted from *Lycium barbarum* L. loaded with isoliquiritigenin to promote spinal cord injury repair based on 3D printed bionic scaffold. Bioeng Transl Med. 2024;9(4): e10646. 10.1002/BTM2.10646. 39036078 10.1002/btm2.10646 PMC11256167

[111] Ishida T, Kawada K, Jobu K, Morisawa S, Kawazoe T, Nishimura S, Akagaki K, Yoshioka S, Miyamura M. Exosome-like nanoparticles derived from *Allium tuberosum* prevent neuroinflammation in microglia-like cells. J Pharm Pharmacol. 2023;75(10):1322–31. 10.1093/JPP/RGAD062. 37390476 10.1093/jpp/rgad062

[112] Wang W, Wang P, Liang Z, Qin Z, Su R, Yin Q, Wang B, Chen J, Zhang Y, Wei X, Huang L, Zhang S, Qi S. Exosome-like nanovesicles derived from *Momordica charantia* ameliorate delayed t-PA thrombolysis-induced hemorrhagic transformation by inhibiting the ONOO−/HMGB1/MMP-9 pathway. J Funct Foods. 2024;114: 106086. 10.1016/j.jff.2024.106086.

[113] Ma Q. Role of Nrf2 in oxidative stress and toxicity. Annu Rev Pharmacol Toxicol. 2013;53(1):401–26. 10.1146/annurev-pharmtox-011112-140320. 23294312 10.1146/annurev-pharmtox-011112-140320 PMC4680839

[114] Kensler TW, Wakabayashi N, Biswal S. Cell survival responses to environmental stresses via the Keap1-Nrf2-ARE pathway. Annu Rev Pharmacol Toxicol. 2007;47(1):89–116. 10.1146/annurev.pharmtox.46.120604.141046. 16968214 10.1146/annurev.pharmtox.46.120604.141046

[115] Cuadrado A, Rojo AI, Wells G, Hayes JD, Cousin SP, Rumsey WL, Attucks OC, Franklin S, Levonen A-L, Kensler TW, Dinkova-Kostova AT. Therapeutic targeting of the NRF2 and KEAP1 partnership in chronic diseases. Nat Rev Drug Discov. 2019;18(4):295–317. 10.1038/s41573-018-0008-x. 30610225 10.1038/s41573-018-0008-x

[116] Cao Y, Zhao Q, Liu F, Zheng L, Lin X, Pan M, Tan X, Sun G, Zhao K. Drug value of *Drynariae Rhizoma* root-derived extracellular vesicles for neurodegenerative diseases based on proteomics and bioinformatics. Plant Signal Behav. 2022. 10.1080/15592324.2022.2129290. 36196516 10.1080/15592324.2022.2129290 PMC9542947

[117] Zhang Y, Lu L, Li Y, Liu H, Zhou W, Zhang L. Response surface methodology optimization of exosome-like nanovesicles extraction from *Lycium ruthenicum* Murray and their inhibitory effects on Aβ-induced apoptosis and oxidative stress in HT22 cells. Foods. 2024;13(20): 3328. 10.3390/foods13203328. 39456390 10.3390/foods13203328 PMC11507227

[118] Zhang Y, Zhang X, Kai T, Zhang L, Li A. *Lycium ruthenicum* Murray derived exosome-like nanovesicles inhibit Aβ-induced apoptosis in PC12 cells via MAPK and PI3K/AKT signaling pathways. Int J Biol Macromol. 2024;277: 134309. 10.1016/j.ijbiomac.2024.134309. 39089544 10.1016/j.ijbiomac.2024.134309

[119] Xu F, Mu J, Teng Y, Zhang X, Sundaram K, Sriwastva MK, Kumar A, Lei C, Zhang L, Liu QM, Yan J, McClain CJ, Merchant ML, Zhang HG. Restoring oat nanoparticles mediated brain memory function of mice fed alcohol by sorting inflammatory Dectin-1 complex into microglial exosomes. Small. 2022. 10.1002/SMLL.202105385. 34897972 10.1002/smll.202105385 PMC8858573

[120] Xu Y, Yan G, Zhao J, Ren Y, Xiao Q, Tan M, Peng L. Plant-derived exosomes as cell homogeneous nanoplatforms for brain biomacromolecules delivery ameliorate mitochondrial dysfunction against Parkinson’s disease. Nano Today. 2024;58: 102438. 10.1016/J.NANTOD.2024.102438.

[121] Elmore S. Apoptosis: a review of programmed cell death. Toxicol Pathol. 2007;35(4):495–516. 10.1080/01926230701320337. 17562483 10.1080/01926230701320337 PMC2117903

[122] Yoon HJ, Won JP, Lee HG, Seo HG. Green onion-derived exosome-like nanoparticles prevent ferroptotic cell death triggered by glutamate: implication for GPX4 expression. Nutrients. 2024;16(19): 3257. 10.3390/nu16193257. 39408223 10.3390/nu16193257 PMC11478619

[123] Bai C, liu J, Zhang X, Li Y, Qin Q, Song H, Yuan C, Huang Z. Research status and challenges of plant-derived exosome-like nanoparticles. Biomed Pharmacother. 2024;174: 116543. 10.1016/j.biopha.2024.116543. 38608523 10.1016/j.biopha.2024.116543

[124] Giraldo E, Lloret A, Fuchsberger T, Viña J. Aβ and tau toxicities in Alzheimer’s are linked via oxidative stress-induced p38 activation: protective role of vitamin E. Redox Biol. 2014;2:873–7. 10.1016/j.redox.2014.03.002. 25061569 10.1016/j.redox.2014.03.002 PMC4099506

[125] Marino G, Calabresi P, Ghiglieri V. Alpha-synuclein and cortico-striatal plasticity in animal models of Parkinson disease; 2022. p. 153–166. 10.1016/B978-0-12-819410-2.00008-4. 10.1016/B978-0-12-819410-2.00008-4 35034731

[126] Ebner K, Singewald N. Individual differences in stress susceptibility and stress inhibitory mechanisms. Curr Opin Behav Sci. 2017;14:54–64. 10.1016/J.COBEHA.2016.11.016.

[127] Cai H, Huang L-Y, Hong R, Song J-X, Guo X-J, Zhou W, Hu Z-L, Wang W, Wang Y-L, Shen J-G, Qi S-H. *Momordica charantia* exosome-like nanoparticles exert neuroprotective effects against ischemic brain injury via inhibiting matrix metalloproteinase 9 and activating the AKT/GSK3β signaling pathway. Front Pharmacol. 2022. 10.3389/fphar.2022.908830. 35814200 10.3389/fphar.2022.908830 PMC9263912

[128] Kim DK, Rhee WJ. Antioxidative effects of carrot-derived nanovesicles in cardiomyoblast and neuroblastoma cells. Pharmaceutics. 2021. 10.3390/pharmaceutics13081203. 34452164 10.3390/pharmaceutics13081203 PMC8400689

[129] He X, Li Y, Deng B, Lin A, Zhang G, Ma M, Wang Y, Yang Y, Kang X. The PI3K / AKT signalling pathway in inflammation, cell death and glial scar formation after traumatic spinal cord injury: mechanisms and therapeutic opportunities. Cell Prolif. 2022;55(9): e13275. 10.1111/cpr.13275. 35754255 10.1111/cpr.13275 PMC9436900

[130] Kim J, Zhu Y, Chen S, Wang D, Zhang S, Xia J, Li S, Qiu Q, Lee H, Wang J. Anti-glioma effect of ginseng-derived exosomes-like nanoparticles by active blood–brain-barrier penetration and tumor microenvironment modulation. J Nanobiotechnology. 2023;21(1): 253. 10.1186/s12951-023-02006-x. 37542285 10.1186/s12951-023-02006-x PMC10401762

[131] Niu W, Xiao Q, Wang X, Zhu J, Li J, Liang X, Peng Y, Wu C, Lu R, Pan Y, Luo J, Zhong X, He H, Rong Z, Fan J-B, Wang Y. A biomimetic drug delivery system by integrating Grapefruit extracellular vesicles and Doxorubicin-loaded Heparin-based nanoparticles for glioma therapy. Nano Lett. 2021;21(3):1484–92. 10.1021/acs.nanolett.0c04753. 33475372 10.1021/acs.nanolett.0c04753

[132] Chen J, Pan J, Liu S, Zhang Y, Sha S, Guo H, Wang X, Hao X, Zhou H, Tao S, Wang Y, Fan J. Fruit-derived extracellular-vesicle-engineered structural droplet drugs for enhanced glioblastoma chemotherapy. Adv Mater. 2023. 10.1002/adma.202304187. 37589312 10.1002/adma.202304187

[133] Zhuang X, Teng Y, Samykutty A, Mu J, Deng Z, Zhang L, Cao P, Rong Y, Yan J, Miller D, Zhang HG. Grapefruit-derived nanovectors delivering therapeutic miR17 through an intranasal route inhibit brain tumor progression. Mol Ther. 2016;24(1):96–105. 10.1038/MT.2015.188. 26444082 10.1038/mt.2015.188 PMC4754550

[134] Barichello T, Collodel A, Hasbun R, Morales R. An Overview of the Blood-Brain Barrier; 2019. p. 1–8. 10.1007/978-1-4939-8946-1_1.

